# The speed of invasion in an advancing population

**DOI:** 10.1007/s00285-023-01989-3

**Published:** 2023-09-12

**Authors:** Anton Bovier, Lisa Hartung

**Affiliations:** 1 Institut für Angewandte Mathematik, University of Bonn, Endenicher Allee 60, 53115 Bonn, Germany; 2 Institut für Mathematik, Johannes Gutenberg-Universität Mainz, Staudingerweg 9, 55099 Mainz, Germany

**Keywords:** F-KPP equations, Invading traits, Travelling waves, Feynman–Kac representation, 60J80, 60G70, 35C07, 92D25

## Abstract

We derive rigorous estimates on the speed of invasion of an advantageous trait in a spatially advancing population in the context of a system of one-dimensional F-KPP equations. The model was introduced and studied heuristically and numerically in a paper by Venegas-Ortiz et al. (Genetics 196:497–507, 2014). In that paper, it was noted that the speed of invasion by the mutant trait is faster when the resident population is expanding in space compared to the speed when the resident population is already present everywhere. We use the Feynman–Kac representation to provide rigorous estimates that confirm these predictions.

## Introduction

The present paper is motivated by an interesting paper by Venegas-Ortiz et al. (2014) that investigates the invasion of a spatially expanding population by a new trait. The classical model for the invasion of a gene in a spatially extended population (Fisher 1937) or the expansion of a population in space (Kolmogorov et al. 1937) is the Fisher–Kolmogorov–Petrovsky–Piscounov (F-KPP) equation, that has been the subject of intense investigation for over 80 years.^1^ The F-KPP equation is a non-linear reaction-diffusion equation that admits travelling wave solutions to which solutions starting with suitable initial conditions converge. This has been known since the early work of Kolmogorov et al. (1937), but has been made both more precise and more general in the seminal book by Bramson (1983).

The model discussed in Venegas-Ortiz et al. (2014) is a system of two coupled equations of the F-KPP type that describes the evolution of a population of two types (traits, alleles,..) that diffuse, compete, and and switch between types. More specifically, they propose the system of equations

1.1 $$\begin{aligned} \partial _t N_A= & {} \frac{1}{2} \partial _{xx} N_A +\alpha N_A(K-N_A-N_B)-\beta N_A+\gamma N_AN_B, \end{aligned}$$

1.2 $$\begin{aligned} \partial _t N_B= & {} \frac{1}{2} \partial _{xx} N_B +\alpha N_B(K-N_A-N_B)+\beta N_A-\gamma N_AN_B. \end{aligned}$$

$N_A,N_B$ represent the masses of traits *A* and *B*, *K* is the carrying capacity, $\alpha ,\beta ,\gamma$ are parameters that satisfy

1.3 $$\begin{aligned} \alpha>\gamma >\beta /K\ge 0. \end{aligned}$$

The different terms in these equations correspond to the following biological mechanisms:

(i) The terms $\partial _{xx} N$ model the spatial diffusion of the population. Note that the diffusion coefficients are the same for both types. This can be seen as biologically plausible, but this choice is mainly done to simplify the mathematical treatment.
(ii) The terms proportional to $\alpha$ describe logistic growth with the quadratic terms corresponding to competitive pressure. Again it is assumed that the pressure exerted by both types and on each type are the same. This again simplifies the mathematics.
(iii) The linear terms $\pm \beta N_A$ can be interpreted as mutation rates from the *A* population to the *B* population. There effect is a net disadvantage of the *A* population.
(iv) The non-linear terms $\pm \gamma N_AN_B$ are interpreted as horizontal gene transfer from the *B*-types to the *A*-types. The idea is that when an *A* individual encounters a *B* individual, the genotype of the *B* individual can be switched to the *A*-type.

The choice of parameters in (1.3) ensures that the a priori disadvantaged *A* type can reemerge in a developed *B*-population and a stable equilibrium with co-existing types exists. The question addressed in Venegas-Ortiz et al. (2014) is to analyse how this effect leads to a hitchhiking of the *A*-type when the *B*-type is spreading in space. The authors of Venegas-Ortiz et al. (2014) make the following interesting and somewhat surprising observation. There are two easily derived travelling waves in the system. First, a population made purely of *B* individuals remains in that state and advances with a speed $v_B$. Second, if *B* has invaded all space, and a *A* population is introduced, there is (with the choice of parameters that ensures the instability of the *B* population against the invasion of *A* individuals) a travelling wave of *A* particles that advances in the background of *B* particles with a speed $v_A<v_B$. If, however, one starts with initial conditions where *A* and *B* particles are present, say in the negative half-line, then the *B* population advances with speed $v_B$ again, but in some parameter range the *A* population advances with a speed $v_c$ that is strictly larger than the speed $v_A$ (and smaller than $v_B$). Somehow, the *A* individuals sense the empty space ahead of the *B*-wave and get attracted to it.

Venegas-Ortiz et al. (2014) derive this result, and precise formulas for the speeds, using local linearisation and matching of solutions. These findings are supported by numerical simulations. In the present paper we derive rigorous estimates on the speeds using the Feynman–Kac representation, originally employed by Bramson (1983) to control the precise speed of convergence to the travelling wave in the original F-KPP equation. It turns out that this point of view not only allows to give rigorous and precise bounds on the solutions of the system of equations, and hence the speeds, but also provides a clear and intuitive explanation for the fact that the empty space ahead of the *B*-wave allows for a faster advance of the *A*-wave. Namely, we will see that this is driven by large, unlikely excursion of the Brownian motion in the Feynman–Kac formula that reach ahead of the front of the *B*-wave. Mathematically, this involves some delicate estimates on probabilities of large excursions of Brownian bridges.

Systems of coupled F-KPP equations have been studied in different contexts in the literature, see e.g. Girardin and Lam (2019), Champneys et al. (1995), Holzer (2012), Holzer and Scheel (2012), Holzer and Scheel (2014), Holzer (2016), Faye and Peltier (2018), Keenan and Cornell (2021) and Lam and Yu (2022). In particular, an analogous result to that in Venegas-Ortiz et al. (2014) and the present paper was derived rigorously in Holzer and Scheel (2014) using analytic methods. Rather recently, there has been interest in such systems in the context of dormancy, see e.g. Blath et al. (2022). Applicable tools depend on the details of the equations. Girardin and Lam (2019) use purely analytic methods involving sub- and super-solutions, while the equations appearing in Champneys et al. (1995) and Blath et al. (2022) allow for a representation in terms of branching Brownian motion and the use of martingale methods. The equations in Venegas-Ortiz et al. (2014), Holzer and Scheel (2014), Holzer (2016) and Faye and Peltier (2018) are particularly nice, as they allow for the use of the Feynman–Kac representation. However, even the introduction of two different diffusion constants seems to spoil this feature, and it seems unclear (albeit interesting) to see how this method can be extended to more general settings.

*Outline* The remainder of this paper is organised as follows. In Sect. 2 we give a precise formulation of the model put forward in Venegas-Ortiz et al. (2014) and explain the special structure of the system that effectively reduces the problem to a time-dependent one-dimensional F-KPP equation. Afterwards we state our main result. Along the way we also recall some background on the standard F-KPP equation that will be needed. In Sect. 3 we present the Feynman–Kac representation, derive some first bounds, and give a heuristic explanation of the main result, based on the Feynman–Kac representation. Section 4 provides the necessary upper and lower bounds on the excursions of Brownian bridges. We compute fairly sharp bounds on the Laplace transforms of these excursions using the Laplace method. Armed with these estimates, we derive upper and lower bounds on solutions from which the wave speed $v_c$ is inferred in Sect. 5. At the end of the paper, in Sect. 6, we discuss our results and point to possible future extensions.

## The F-KPP equations

It is convenient to introduce the total population mass $N_T\equiv N_A+N_B$ and to write the Eqs. (1.1) and (1.2) in the the form

2.1 $$\begin{aligned} \partial _t N_T= & {} \frac{1}{2} \partial _{xx} N_T +\alpha N_T(K-N_T), \end{aligned}$$

2.2 $$\begin{aligned} \partial _t N_A= & {} \frac{1}{2} \partial _{xx} N_A +\alpha N_A(K-N_T)-\beta N_A+\gamma N_A(N_T-N_A). \end{aligned}$$

We see that $N_T$ satisfies an autonomous F-KPP equation. Effectively, the second equation is a F-KPP equation with time dependent reaction rates. This structure is crucial for our analysis building on the Feynman–Kac formula. Equations of a similar structure have been also been studied in Holzer (2016) and Faye and Peltier (2018). It is furthermore convenient to eliminate the parameters *K* and $\alpha$ by rescaling. We define

2.3 $$\begin{aligned} v(t,x)\equiv & {} \frac{1}{K} N_T(t/(\alpha K), x/\sqrt{\alpha K}), \end{aligned}$$

2.4 $$\begin{aligned} w(t,x)\equiv & {} \frac{1}{K} N_A(t/(\alpha K), x/\sqrt{\alpha K}). \end{aligned}$$

Then *v* and *w* solve

2.5 $$\begin{aligned} \partial _t v= & {} \frac{1}{2}\partial _{xx} v+v(1-v), \end{aligned}$$

2.6 $$\begin{aligned} \partial _t w= & {} \frac{1}{2}\partial _{xx} w +\left( 1-{\tilde{\beta }}-(1-{\tilde{\gamma }})v-{\tilde{\gamma }}w\right) w. \end{aligned}$$

where ${\tilde{\beta }}=\beta /(\alpha K)$ and ${\tilde{\gamma }}=\gamma /\alpha$. Note that $1>{\tilde{\gamma }}>{\tilde{\beta }}>0$.

Note that the system of equations has four spatially constant fixpoints:

(i) $$v=0, w=0$$,
(ii) $$v=0, w=(1-{\tilde{\beta }})/{\tilde{\gamma }}$$,
(iii) $$v=1,w=0$$,
(iv) $$v=1,w=1-{\tilde{\beta }}/{\tilde{\gamma }}$$.

The fixpoint (ii) is unphysical, since it corresponds to a negative mass for the population *B*. The fixpoints (i) and (iii) are unstable, and (iv) is the stable fixpoint.

The behaviour of *v* is well-known from Bramson’s work (Bramson 1983), so solving for *w* amounts to solve the F-KPP equation with time dependent coefficients. A particularly simple situation arises if we choose initial conditions such that $v(0,x)=1$, for all $x\in {\mathbb {R}}$. In that case *w* solves the F-KPP equation

2.7 $$\begin{aligned} \partial _t w=\frac{1}{2}\partial _{xx} w +\left( {\tilde{\gamma }}-{\tilde{\beta }}-{\tilde{\gamma }}w\right) w. \end{aligned}$$

In this case, with suitable initial conditions (e.g. Heaviside), *w* converges to a travelling wave solution that moves with speed $\sqrt{2({\tilde{\gamma }}-{\tilde{\beta }})}$. A more interesting situation arises if the initial conditions are such that *v*(0, *x*) decays rapidly at $+\infty$ and *w*(0, *x*) is non-zero. In that case, Venegas-Ortiz et al. (2014) observed that the *w*-wave follows behind the *v*-wave, but moves faster than it would in a fully established population. Recall that the standard F-KPP equation (2.5) admits travelling wave solutions

2.8 $$\begin{aligned} v(t,x+\lambda t)=\omega (x), \end{aligned}$$

where $\omega$ solves the ode

2.9 $$\begin{aligned} \frac{1}{2}\partial _{xx} \omega +\lambda \partial _x \omega +\omega (1-\omega )=0, \end{aligned}$$

for all speeds $\ge \sqrt{2}$. It was shown by Kolmogorov et al. (1937) that (2.9) has a unique solution up to translations such that $\lim _{x\downarrow -\infty }\omega (x)=1$ and $\lim _{x\uparrow \infty }\omega (x)=0$. We are only interested in the case $\lambda =\sqrt{2}$, since solutions with initial condition that converge rapidly to zero at infinity, and in particular with Heaviside initial conditions, converge to travelling waves with this speed (see Bramson (1983) for more details).

We pick the solution for which $\omega (0)=1/2$. Lalley and Sellke (1987) derived the probabilistic representation

2.10 $$\begin{aligned} 1-\omega (x)= {\mathbb { E}}\left[ {\mathrm e}^{-ZC{\mathrm e}^{-\sqrt{2\pi }x}}\right] , \end{aligned}$$

where *Z* is a random variable, the limit of the so-called *derivative martingale*, and *C* is a constant such that

2.11 $$\begin{aligned} {\mathbb { E}}\left[ {\mathrm e}^{-ZC}\right] =\frac{1}{2}. \end{aligned}$$

Clearly, if *v* solves (2.5) with initial condition $v(0,x) = \omega (x+a)$, then $v(t,x)=\omega (x+a-\sqrt{2} t)$. It is known that

2.12 $$\begin{aligned} \omega (x) \sim C x {\mathrm e}^{-\sqrt{2} x},\quad \text {as}\; x\uparrow +\infty , \end{aligned}$$

and

2.13 $$\begin{aligned} \omega (x) \sim 1-c \textrm{e}^{-(2-\sqrt{2})x},\quad \text {as}\; x\downarrow -\infty . \end{aligned}$$

[(2.12) is due to Bramson, (2.13) is proven in the first arXiv version of Arguin et al. (2011)]. Bramson has shown that for any initial conditions that decay faster than ${\mathrm e}^{-\sqrt{2}x}$ at $+\infty$,

2.14 $$\begin{aligned} v(t,x-m(t)) \rightarrow \omega (x), \end{aligned}$$

uniformly in *x*, as $t\uparrow \infty$, where

2.15 $$\begin{aligned} m(t)=\sqrt{2} t-\frac{3}{2\sqrt{2}}\ln t. \end{aligned}$$

It will be convenient to analyse the system (2.5), (2.6) with initial conditions $v(0,x)=\omega (x+a)$ and $w(0,x) =(1-{\tilde{\beta }}/{\tilde{\gamma }}) \mathbbm {1}_{x\le 0}$. With this choice, our problem is reduced to studying the scalar equation

2.16 $$\begin{aligned} \partial _t w(t,x)= & {} \frac{1}{2}\partial _{xx} w(t,x)\nonumber \\{} & {} +\left( 1-{\tilde{\beta }}-(1-{\tilde{\gamma }})\omega \left( x+a-\sqrt{2}t\right) -{\tilde{\gamma }}w(t,x) \right) w(t,x),\nonumber \\ \end{aligned}$$

with initial condition $w(0,x) =(1-{\tilde{\beta }}/{\tilde{\gamma }}) \mathbbm {1}_{x\le 0}$.

Our main result is the following.

### Theorem 1.1

Let $$a\in {\mathbb {R}}_+$$. Let

2.17 $$\begin{aligned} u_c\equiv \max \left( \sqrt{2}-\frac{{\tilde{\beta }}}{\sqrt{2}{\tilde{\gamma }}} \left( 1+\sqrt{1-{\tilde{\gamma }}}\right) ,\sqrt{2\left( {\tilde{\gamma }}-{\tilde{\beta }}\right) }\right) \end{aligned}$$

Then for all $\delta >0$ sufficiently small there exist constants $C_1,C_2>0$ such that

2.18 $$\begin{aligned} w(t, u_ct-C_1\ln t)>\delta \end{aligned}$$

and

2.19 $$\begin{aligned} w(t, u_ct+C_2\ln t+z)<1/t, \quad \forall z>0, \end{aligned}$$

for all *t* large enough.

### Remark

Note that $u_c$ is strictly larger than $\sqrt{2\left( {\tilde{\gamma }}-{\tilde{\beta }}\right) }$ for ${\tilde{\beta }}$ small enough. Notice that Venegas-Ortiz et al. derive in Venegas-Ortiz et al. (2014) a rather complicated looking equation, (Eq. 8), and a simpler one (Eq. 9), obtained by expanding in ${\tilde{\beta }}$. Our results show that the second version is exact, provided ${\tilde{\beta }}$ is such that

2.20 $$\begin{aligned} \sqrt{2}-\frac{{\tilde{\beta }}}{\sqrt{2}{\tilde{\gamma }}} \left( 1+\sqrt{1-{\tilde{\gamma }}}\right) \ge \sqrt{2\left( {\tilde{\gamma }}-{\tilde{\beta }}\right) }, \end{aligned}$$

while the first seems incorrect. This is also in agreement with the finding in Girardin and Lam (2019). An analogous result on an accelerated speed in a slightly different system of equations was derived by purely analytic methods by Holzer and Scheel (2014), Lemma 11.

### Remark

Note that in fact the result of Theorem 2.1 does not depend on the choice of *a* in the initial condition. This is not surprising as a finite shift of the initial condition does not affect the large time asymptotic of the solutions.

The remainder of this paper is devoted to proving Theorem 2.1. In the process, we will derive precise bounds on the behaviour of the solutions.

## The Feynman–Kac representation

Bramson’s analysis of the F-KPP equation (Bramson 1983) is based on the Feynman–Kac representation. We will do the same for the Eq. (2.16).

### The representation and elementary bounds

#### Lemma 1.2

The solution of (2.16) satisfies the equation

3.1 $$\begin{aligned} w(t,x)= & {} {\mathbb { E}}_x\bigg [\exp \bigg (\int _0^t \Big (1-{\tilde{\beta }}-(1-{\tilde{\gamma }})\omega \left( B_s+a-\sqrt{2}(t-s)\right) \nonumber \\{} & {} -{\tilde{\gamma }}w(t-s, B_s) \Big )ds\bigg )w(0,B_t)\bigg ], \end{aligned}$$

where *B* is a Brownian motion starting in *x*.

#### Proof

The proof is identical to the one in Bramson (1983). $\square$

It is convenient to express the Brownian motion *B* in terms of its endpoint $B_t$ and a Brownian bridge

3.2 $$\begin{aligned} {{\mathfrak {z}}}_{x,B_t}^t(s) = x+\frac{s}{t} (B_t-x) + {{\mathfrak {z}}}_{0,0}^t, \end{aligned}$$

from *x* to $B_t$. Here ${{\mathfrak {z}}}_{0,0}^t$ is a Brownian bridge from 0 to 0 in time *t*. Note that the bridge is independent of $B_t$. This leads to the following reformulation of (3.1).

#### Lemma 1.3

The solution of (2.16) satisfies

3.3 $$\begin{aligned} w(t,x)= & {} \frac{1}{\sqrt{2 \pi t}}\int _{-\infty }^\infty dy {\mathrm e}^{-\frac{(x-y)^2}{2t}} w(0,y)\nonumber \\{} & {} \times \, {\mathbb { E}}\bigg [\exp \Bigg (\int _0^t \Bigg (1-{\tilde{\beta }}-(1-{\tilde{\gamma }})\omega \left( {{\mathfrak {z}}}^t_{x,y}(s)+a-\sqrt{2}(t-s)\right) \nonumber \\{} & {} \quad -{\tilde{\gamma }}w\left( t-s, {{\mathfrak {z}}}_{x,y}^t(s)\right) \Bigg )ds\Bigg )\Bigg ]\nonumber \\= & {} \frac{1}{\sqrt{2 \pi t}}\int _{-\infty }^\infty dy {\mathrm e}^{-\frac{(x-y)^2}{2t}} w(0,y)\nonumber \\{} & {} \times \,{\mathbb { E}}\Biggl [\exp \Biggl (\int _0^t \biggl (1-{\tilde{\beta }}-(1-{\tilde{\gamma }})\omega \left( x{\textstyle {t-s\over t}} +{\textstyle {s\over t}} y +{{\mathfrak {z}}}^t_{0,0}(s)+a-\sqrt{2}(t-s)\right) \nonumber \\{} & {} -\,{\tilde{\gamma }}w\left( t-s, x{\textstyle {t-s\over t}} +{\textstyle {s\over t}} y +{{\mathfrak {z}}}_{0,0}^t(s)\right) \biggr )ds\Biggr )\Biggr ], \end{aligned}$$

where ${\mathbb { E}}$ now refers to the expectation with respect to the Brownian bridges ${{\mathfrak {z}}}_{x,y}^t$ resp. ${{\mathfrak {z}}}_{0,0}^t$.

#### Proof

Elementary.$$\square $$

The fact that $0\le \omega \le 1$ and $0\le w\le 1-{\tilde{\gamma }}/{\tilde{\beta }}$ yields the first bounds.

#### Lemma 1.4

The solution of (2.16) satisfies

3.4 $$\begin{aligned} w(t,x)\le \frac{1}{\sqrt{2 \pi t}}\int _{-\infty }^\infty dy {\mathrm e}^{-\frac{(x-y)^2}{2t}} w(0,y) {\mathrm e}^{(1-{\tilde{\beta }})t}. \end{aligned}$$

and

3.5 $$\begin{aligned} w(t,x)\ge \frac{1}{\sqrt{2 \pi t}}\int _{-\infty }^\infty dy {\mathrm e}^{-\frac{(x-y)^2}{2t}} w(0,y). \end{aligned}$$

For Heaviside initial conditions, this implies

3.6 $$\begin{aligned} \sqrt{\frac{t}{2\pi }} \frac{{\mathrm e}^{-\frac{x^2}{2t}}}{x}\left( 1-O (t/ x^2)\right) \le \frac{w(t,x)}{(1-{\tilde{\beta }}/{\tilde{\gamma }})} \le \sqrt{\frac{t}{2\pi }} \frac{{\mathrm e}^{-\frac{x^2}{2t}+(1-{\tilde{\beta }})t}}{x}. \end{aligned}$$

#### Proof

Equations (3.4) and (3.5) are immediate from the bounds on $\omega$ and *w* mentioned above. (3.6) follows from the standard Gaussian tail estimates, see, e.g. Leadbetter et al. (1983). $\square$

### First heuristics

Since the term involving $\omega$ is explicit, we can improve the upper bound (3.4) as follows.

3.7 $$\begin{aligned} w(t,x)\le & {} {\textstyle {(1-{\tilde{\beta }}/{\tilde{\gamma }})\over \sqrt{2 \pi t}}}\int _{-\infty } dy {\mathrm e}^{-\frac{(x-y)^2}{2t}} \nonumber \\{} & {} \times \, {\mathbb { E}}\Biggl [\exp \Biggl (\int _0^t \biggl (1-{\tilde{\beta }}-(1-{\tilde{\gamma }})\omega \left( x{\textstyle {t-s\over t}} +{\textstyle {s\over t}} y +{{\mathfrak {z}}}^t_{0,0}(s)\right. \nonumber \\{} & {} \left. +\,a-\sqrt{2}(t-s)\right) \biggr )ds\Biggr )\Biggr ]. \end{aligned}$$

Since $w\le \omega$, we also have the lower bound

3.8 $$\begin{aligned} w(t,x)&\ge {\textstyle {(1-{\tilde{\beta }}/{\tilde{\gamma }})\over \sqrt{2 \pi t}}}\int _{-\infty }^0 dy {\mathrm e}^{-\frac{(x-y)^2}{2t}} \nonumber \\&\quad \times \, {\mathbb { E}}\Biggl [\exp \Biggl (\int _0^t \biggl (1-{\tilde{\beta }}-\omega \left( x{\textstyle {t-s\over t}} +{\textstyle {s\over t}} y +{{\mathfrak {z}}}^t_{0,0}(s)+a-\sqrt{2}(t-s)\right) \biggr )ds\Biggr )\Biggr ]. \end{aligned}$$

To see how we can use these bounds, let us first ignore the possible excursions of the Brownian bridge and simply set ${{\mathfrak {z}}}_{0,0}^t(s)=0$. We want to see where *w*(*t*, *x*) drops from 1 to zero. From (3.6) we already know that this must happen before $x=\sqrt{2(1-{\tilde{\beta }})} t$. Now assume that for some $u\le \sqrt{2(1-{\tilde{\beta }})}$, $w(t,ut +z)\le \epsilon$, for all $z\ge 0$. Then, for $z\ge 0$ independent of *t*,

3.9 $$\begin{aligned} w(t,ut+z)\ge & {} {\textstyle {(1-{\tilde{\beta }}/{\tilde{\gamma }})\over \sqrt{2 \pi t}}}\int _{-\infty }^0dy {\mathrm e}^{-\frac{(ut+z-y)^2}{2t}}\nonumber \\{} & {} \times \exp \Biggl (\int _0^t \biggl (1-{\tilde{\beta }}-\omega \left( (u-\sqrt{2})(t-s) +z {\textstyle {t-s\over t}}+{\textstyle {s\over t}} y +a\right) \nonumber \\{} & {} -\,{\tilde{\gamma }}w\biggl (t-s, u(t-s)+z{\textstyle {t-s\over t}}+{\textstyle {s\over t}} y \biggr )ds\Biggr )\nonumber \\\ge & {} {\textstyle {(1-{\tilde{\beta }}/{\tilde{\gamma }})\over \sqrt{2 \pi t}}}\int _{-\infty }^0dy {\mathrm e}^{-\frac{(ut+z-y)^2}{2t}} \exp \left( t ({\tilde{\gamma }}-{\tilde{\beta }}- \epsilon )\right) \nonumber \\\ge & {} {\textstyle {(1-{\tilde{\beta }}/{\tilde{\gamma }})\over \sqrt{2 \pi t}u}} {\mathrm e}^{-\frac{u^2t}{2} - uz -z^2/(2t)+ t ({\tilde{\gamma }}-{\tilde{\beta }}- \epsilon )}\sim {\mathrm e}^{-\frac{u^2t}{2}+ t ({\tilde{\gamma }}-{\tilde{\beta }}- \epsilon )}, \end{aligned}$$

which tends to infinity if $u<\sqrt{2({\tilde{\gamma }}-{\tilde{\beta }})}$. Hence, the hypothesis can only be true for $u\ge \sqrt{2({\tilde{\gamma }}-{\tilde{\beta }})}$. On the other hand, if $\sqrt{2}>u>\sqrt{2({\tilde{\gamma }}-{\tilde{\beta }})}$, we get the corresponding upper bound

3.10 $$\begin{aligned} w(t,ut+z) \le {\textstyle {(1-{\tilde{\beta }}/{\tilde{\gamma }})\over \sqrt{2 \pi t}u}} {\mathrm e}^{-\frac{u^2t}{2} - uz -z^2/(2t)+ t ({\tilde{\gamma }}-{\tilde{\beta }})}, \end{aligned}$$

which is decaying exponentially with *t*. This suggests a wave moving at speed $u_0=\sqrt{2({\tilde{\gamma }}-{\tilde{\beta }})}$, which is the speed we obtain if $v(0,x)\equiv 1$. This shows that the only way to move faster is to exploit the possibility of the Brownian bridge to make a forward excursion out of the region where $\omega =1$.

### Improved heuristics on the wave speed

First, note that in (3.3) *y* is negative, so that we cannot gain anything from it and pretend that it is equal to zero in this subsection. To simplify the heuristics we also set $a=0$. Moreover, as we are analysing the possible gain in $\omega$ by large Brownian bridge excursions to areas where $\omega$ is small, we will ignore *w* (which is always way smaller than $\omega$) in (3.3). Hence, we are left with estimating

3.11 $$\begin{aligned} \frac{1}{\sqrt{2 \pi t}} {\mathrm e}^{-\frac{x^2}{2t}} {\mathbb { E}}\Biggl [\exp \Biggl (\int _0^t \biggl (1-{\tilde{\beta }}-(1-{\tilde{\gamma }})\omega \left( x{\textstyle {t-s\over t}} +{{\mathfrak {z}}}^t_{0,0}(s)-\sqrt{2}(t-s)\right) \biggr )ds\Biggr )\Biggr ]. \end{aligned}$$

For our heuristics we approximate $\omega$ by

3.12 $$\begin{aligned} \omega \left( x{\textstyle {t-s\over t}} +{{\mathfrak {z}}}^t_{0,0}(s)-\sqrt{2}(t-s)\right) \approx \mathbbm {1}_{x{\textstyle {t-s\over t}} +{{\mathfrak {z}}}^t_{0,0}(s)-\sqrt{2}(t-s)\le 0}. \end{aligned}$$

Hence, to further estimate the expectation in (3.11) we need an estimate on the time during which the indicator function takes the value 0. To this end, let

3.13 $$\begin{aligned} T_t\equiv \int _0^t \mathbbm {1}_{{{\mathfrak {z}}}_{0,0}^t(s)\ge \alpha (t- s)}ds, \end{aligned}$$

with $\alpha =\sqrt{2}-x/t$, be the time the Brownian bridge spends above a line with slope $\alpha$. Note that (3.11) is then approximately equal to

3.14 $$\begin{aligned} \frac{1}{\sqrt{2 \pi t}} {\mathrm e}^{-\frac{x^2}{2t}} {\mathrm e}^{\left( {\tilde{\gamma }}-{\tilde{\beta }}\right) t} {\mathbb { E}}\left[ {\mathrm e}^{(1-{\tilde{\gamma }})T_t}\right] . \end{aligned}$$

Next, on the exponential scale

3.15 $$\begin{aligned} {\mathbb {P}}(T_t>S)\approx & {} {\mathbb {P}}(T_t\approx S)\approx {\mathbb {P}}\left( {{\mathfrak {z}}}^t_{0,0}(S)\approx \left( \sqrt{2} -x/t\right) S\right) \nonumber \\= & {} \sqrt{{\textstyle {t\over 2\pi (t-S)S}}} {\mathrm e}^{-\frac{\left( \sqrt{2} -x/t\right) ^2St}{2(t-S)}}, \end{aligned}$$

where we used that heuristically the cheapest way to realise the event $\{T_t>S\}$ is to stay above this line up to roughly time *S*. This probability is roughly dominated by the event to be essentially on the line at time *S*. As we gain a factor $(1-{\tilde{\gamma }})$ (on the exponential scale) as long as the Brownian bridge is above the line with slope $(\sqrt{2} -x/t)$, to find the dominating event in the expectation in (3.11) we need to find the optimal $S^*$, namely

3.16 $$\begin{aligned} S^*\equiv \text{ argmax}_S \left( -S {\textstyle {t\alpha ^2\over 2(t-S)}} + (1-{\tilde{\gamma }})S\right) . \end{aligned}$$

By differentiating the right-hand side of (3.16), we see that

3.17 $$\begin{aligned} S^*= t\left( 1-{\textstyle {\sqrt{2}-x/t\over \sqrt{2(1-{\tilde{\gamma }})}}}\right) . \end{aligned}$$

Now, we distinguish two cases.

1. If $S^*$ is positive, we plug this back into (3.14). Then the exponent in (3.14) is to leading order equal to
  3.18 $$\begin{aligned}{} & {} -{\textstyle {x^2\over 2t}}+ t(1-{\tilde{\beta }})-t\sqrt{2(1-{\tilde{\gamma }})}\left( \sqrt{2}-x/t\right) +{\textstyle {\left( \sqrt{2}-x/t\right) ^2\over 2}}t\nonumber \\{} & {} \quad = -{\tilde{\beta }}t+2t(1-\sqrt{1-{\tilde{\gamma }}})- \left( \sqrt{2}-\sqrt{2(1-{\tilde{\gamma }})}\right) x. \end{aligned}$$
  To see where *w* starts to decay to 0, we need to see for which *x* (3.18) is equal to zero (hence its exponential is of order 1). This leads to
  3.19 $$\begin{aligned} x_1^* ({\tilde{\beta }})=\sqrt{2}\left( 1-{\textstyle {{\tilde{\beta }}\over 2{\tilde{\gamma }}}}\left( 1+\sqrt{1-{\tilde{\gamma }}}\right) \right) t. \end{aligned}$$
2. If $S^*\le 0$ in (3.17), we cannot gain anything from the Brownian bridge excursion into areas where $\omega \approx 0$ and always have $\omega =1$. And thus the exponent in (3.11) is approximately
  3.20 $$\begin{aligned} -{\textstyle {x^2\over 2t}}+({\tilde{\gamma }}-{\tilde{\beta }}), \end{aligned}$$
  which is of order one for
  3.21 $$\begin{aligned} x_2^*({\tilde{\beta }})=\sqrt{2({\tilde{\gamma }}-{\tilde{\beta }})}t. \end{aligned}$$

We can summarise (3.18) and (3.20) by

3.22 $$\begin{aligned} w(t,ut)\approx & {} \exp \left( -t\left( {\textstyle {u^2\over 2}}-\left( {\tilde{\gamma }}-{\tilde{\beta }}\right) \right. \right. \nonumber \\{} & {} \left. \left. -\,{\textstyle {1\over 2}}{\left( \sqrt{2}-u-\sqrt{2\left( 1-{\tilde{\gamma }}\right) }\right) ^2}\mathbbm {1}_{u>\sqrt{2}\left( 1-\sqrt{1-{\tilde{\gamma }}}\right) }\right) \right) . \end{aligned}$$

The exponent is zero if $ut=x^*_2({\tilde{\beta }})$ and $u\le \sqrt{2}\left( 1-\sqrt{1-{\tilde{\gamma }}}\right)$ or if $ut=x^*_1({\tilde{\beta }})$ and $u>\sqrt{2}\left( 1-\sqrt{1-{\tilde{\gamma }}}\right)$. Seeing $x_1^*({\tilde{\beta }})$ as a function of ${\tilde{\beta }}$, we observe that it is decreasing in ${\tilde{\beta }}$ and there is exactly one critical value ${\tilde{\beta }}^*_1$ such that

3.23 $$\begin{aligned} x_1^*({\tilde{\beta }}^*_1)= \sqrt{2}\left( 1-\sqrt{1-{\tilde{\gamma }}}\right) t. \end{aligned}$$

Namely,

3.24 $$\begin{aligned} {\tilde{\beta }}_1^*=2\left( {\tilde{\gamma }}+\sqrt{1-{\tilde{\gamma }}}-1\right) . \end{aligned}$$

Similarly, seeing $x_2^*({\tilde{\beta }})$ as a function if ${\tilde{\beta }}$ we observe that it is decreasing in ${\tilde{\beta }}$ and there is exactly one critical value ${\tilde{\beta }}^*_2$ such that

3.25 $$\begin{aligned} x_2^*({\tilde{\beta }}^*_2)= \sqrt{2}\left( 1-\sqrt{1-{\tilde{\gamma }}}\right) t. \end{aligned}$$

Namely,

3.26 $$\begin{aligned} {\tilde{\beta }}_2^*=2\left( {\tilde{\gamma }}+\sqrt{1-{\tilde{\gamma }}}-1\right) ={\tilde{\beta }}_1^*. \end{aligned}$$

As the two critical values for ${\tilde{\beta }}$ are the same, this suggests that for ${\tilde{\beta }}>{\tilde{\beta }}_1^*$ the speed of the wave equals $x_2^*/t$ and increases continuously to $x_1^*$ for ${\tilde{\beta }}<{\tilde{\beta }}_1^*$. This will be made rigorous in the following sections.

## Brownian bridge estimates

In this section we provide the key input about Brownian bridges that is needed to make the heuristics above rigorous.

### Probabilities of excursions

**Fig. 1:**
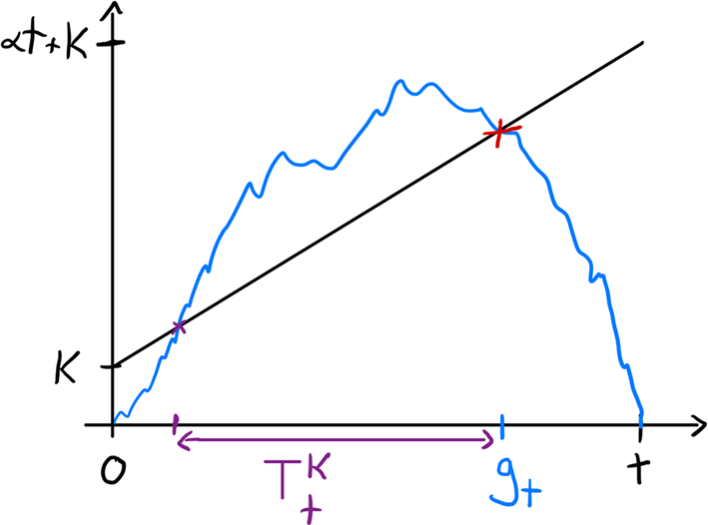
Schematic picture of the Brownian bridge spending time $T^K_t$ above the line $\alpha s+K$

As $\omega$ is not exactly an indicator function, the key question is to know the distribution of the time a Brownian bridge ${{\mathfrak {z}}}_{0,0}^t$ spends well above and well below a line $(\sqrt{2}-u)(t-s)$, $0\le s\le t$. Define, for $\alpha \equiv \sqrt{2}-u$ fixed, for $K\in {\mathbb {R}}$, (see Fig. 1)

4.1 $$\begin{aligned} T^{K}_t\equiv \int _0^t \mathbbm {1}_{{{\mathfrak {z}}}_{0,0}^t(s)\ge \alpha (t- s)+ K}ds. \end{aligned}$$

Note that ${{\mathfrak {z}}}^t_{0,0}(s)$ has the same law as ${{\mathfrak {z}}}^t_{0,0}(t-s)$, and so we can replace $T_t^{K}$ by

4.2 $$\begin{aligned} T^{K}_t= \int _0^t \mathbbm {1}_{{{\mathfrak {z}}}_{0,0}^t(s)\ge \alpha s+ K}ds, \end{aligned}$$

for convenience. The following theorem provides precise tail asymptotic for $T^{K}_t$.

#### Theorem 1.5

Let ${{\mathfrak {z}}}_{0,0}^t$ be a Brownian bridge from zero to zero in time *t*. Let $\alpha >0$ and $T_t$ defined in (4.1). Then, for $0<s\le 1$,

4.3 $$\begin{aligned}{} & {} {\mathbb {P}}\left( T^K_t> st\right) \nonumber \\{} & {} \quad = t^{-3/2}\alpha \sqrt{{\textstyle {1\over 2\pi s^3(1-s)^3}}} {\mathrm e}^{-\frac{t\alpha ^2s}{2(1-s)}-\frac{\alpha K}{1-s}} \nonumber \\{} & {} \qquad \times {\left\{ \begin{array}{ll} \left( {\textstyle {2(1-s)^2\over \alpha ^2}}\right) ^2 {2} \left( 1+o(1)\right) ,&{}\;\textrm{if }\; K=0, \\ \left( {\textstyle {2(1-s)^2\over \alpha ^2}}\right) ^{3/2} {\textstyle {\sqrt{2}K\over \sqrt{\pi }}} \left( 1+o(1)\right) ,&{}\;\textrm{if }\; K<0, \end{array}\right. } \end{aligned}$$

and

4.4 $$\begin{aligned} {\mathbb {P}}\left( T^K_t>st\right)= & {} t^{-3/2} K(\sqrt{\pi }-1) \nonumber \\{} & {} \times \sqrt{{\textstyle { 1-s\over 2\pi s^3}}} {\mathrm e}^{-\frac{t\alpha ^2s}{2(1-s)}-\frac{\alpha K(1+\sqrt{2})}{1-s}} \left( {\textstyle {\sqrt{2}K\alpha \over 1-s}}+1\right) (1+o(1)), \end{aligned}$$

if $$K>0$$.

#### Proof

To start, we define $g_t$ as the last time the Brownian bridge ${{\mathfrak {z}}}_{0,0}^t$ is above the line $\alpha s+K$, (see Fig. 1)

4.5 $$\begin{aligned} g_t\equiv \sup \left\{ u\le t: {{\mathfrak {z}}}_{0,0}^t (u)\ge \alpha u+K\right\} . \end{aligned}$$

Then

4.6 $$\begin{aligned} {\mathbb {P}}\left( T^K_t>S\right)= & {} {\mathbb {P}}\left( \int _0^{g_t} \mathbbm {1}_{{{\mathfrak {z}}}_{-K,0}^{g_t}(u)\ge 0}du\ge S\right) \nonumber \\= & {} {\mathbb { E}}\left[ {\mathbb {P}}\left( \int _0^{g_t} \mathbbm {1}_{{{\mathfrak {z}}}_{-K,0}^{g_t}(u)\ge 0}du\ge S\big | g_t\right) \right] \end{aligned}$$

The conditional probability in (4.6) is known (Pechtl 1999, 2020). A more convenient formula is given in Aurzada and Schickentanz (2022), see Eq. (7) therein. For our setting this yields

4.7 $$\begin{aligned}{} & {} {\mathbb {P}}\left( \int _0^{g_t} \mathbbm {1}_{{{\mathfrak {z}}}_{-K,0}^{g_t}(u)\ge 0}du\ge S\big | g_t\right) \equiv \phi _K(g_t,S) \nonumber \\{} & {} \quad = {\left\{ \begin{array}{ll}-2\left( {\textstyle {S\over g_t}}\left( 1-{\textstyle {K^2\over g_t}}\right) -1\right) \Phi \left( {\textstyle {-K\sqrt{S}\over \sqrt{g_t(g_t-S)}}}\right) -{\textstyle {K\sqrt{2S(g_t-S)}\over \sqrt{\pi g_t^3}}}{\mathrm e}^{-\frac{K^2S}{2g_t(g_t-S)}}, &{}\;\hbox {if}\; K\ge 0,\\ 1+2\left( {\textstyle {g_t-S\over g_t}}\left( 1-{\textstyle {K^2\over g_t}}\right) -1\right) \Phi \left( {\textstyle {K\sqrt{g_t-S}\over \sqrt{g_tS}}}\right) -{\textstyle {K\sqrt{2S(g_t-S)}\over \sqrt{\pi g_t^3}}}{\mathrm e}^{-\frac{K^2(g_t-S)}{2g_tS}}, &{}\;\hbox {if}\; K\le 0, \end{array}\right. }\nonumber \\ \end{aligned}$$

where $\Phi$ is the error function. Note that for $K=0$, this simplifies to

4.8 $$\begin{aligned} \phi _0(g_t,S)=2\left( 1-{\textstyle {S\over g_t}}\right) \Phi (0)=\left( 1-{\textstyle {S\over g_t}}\right) , \end{aligned}$$

which recovers the result that the time spent by a Brownian bridge from 0 to 0 in time $g_t$ above 0 is uniformly distributed on $[0,g_t]$.

Next we need to control the distribution of $g_t$. Fortunately, this can be recovered from known results by Beghin and Orsingher (1999).

#### Lemma 1.6

With the notation above,

4.9 $$\begin{aligned} {\mathbb {P}}\left( g_t\ge q\right) ={\mathrm e}^{-\frac{2K(\alpha t+K)}{t}} \Phi \left( -{\textstyle {(\alpha tq+K(2q-t))\over \sqrt{qt(t-q)}}}\right) +1- \Phi \left( {\textstyle {(\alpha tq)+Kt)\over \sqrt{qt(t-q)}}}\right) . \end{aligned}$$

**Fig. 2:**
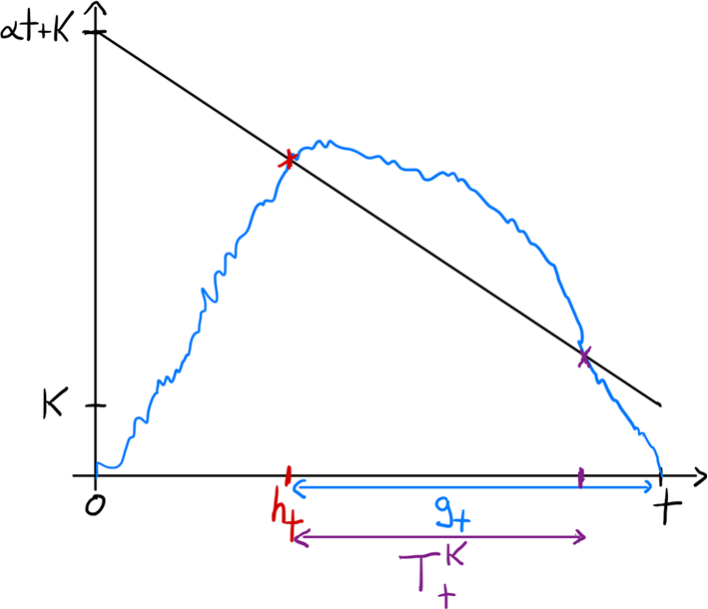
Schematic picture of the Brownian bridge in reversed time

#### Proof

Looking back in time, we see that we can also interpret $g_t$ as

4.10 $$\begin{aligned} g_t=t-\inf \left\{ s>0: {{\mathfrak {z}}}_{0,0}^t(t-s)=\alpha (t-s)+K\right\} . \end{aligned}$$

By time reversal, this has the same law as $t-h_t$ where

4.11 $$\begin{aligned} h_t= & {} \inf \left\{ s>0: {{\mathfrak {z}}}_{0,0}^t(s) = \alpha (t-s)+K \right\} \nonumber \\= & {} \inf \left\{ s>0: {{\mathfrak {z}}}_{0,\alpha t}^t(s) = \alpha t+K \right\} . \end{aligned}$$

The latter probability can be computed using a result by Beghin and Orsingher (1999) (Lemma 2.1). It yields that, for $\alpha t+K>0$,

4.12 $$\begin{aligned} {\mathbb {P}}\left( h_t\le r\right) = {\mathrm e}^{-\frac{2K(\alpha t+K)}{t}} \Phi \left( -{\textstyle {(\alpha t(t-r)+K(t-2r))\over \sqrt{rt(t-r)}}}\right) +1- \Phi \left( {\textstyle {(\alpha t(t-r)+Kt)\over \sqrt{rt(t-r)}}}\right) .\qquad \end{aligned}$$

If $\alpha (t-r)+K\le 0$, then this probability is equal to one. Note that, in particular,

4.13 $$\begin{aligned} {\mathbb {P}}\left( h_t\le t\right) ={\left\{ \begin{array}{ll} {\mathrm e}^{-\frac{2K(\alpha t+K)}{t}},&{}\;\textrm{if}\; K>0,\\ 1,&{}\;\textrm{if}\; K\le 0. \end{array}\right. } \end{aligned}$$

Note that the term in the second line in (4.12) is (asymptotically equal) and smaller than

4.14 $$\begin{aligned} \sqrt{{\textstyle {r(t-r)\over 2\pi (\alpha (t-r)+K)^2t}}} {\mathrm e}^{-\frac{\alpha ^2t(t-r)}{2r}-\frac{K\alpha t}{r}-\frac{K^2t}{2r(t-r)}}. \end{aligned}$$

If $\alpha t(t-r)+K(t-2r)>0$, the first term in (4.12) is asymptotically equal to and smaller than

4.15 $$\begin{aligned} {\mathrm e}^{-\frac{2K(\alpha t+K)}{t}} \sqrt{{\textstyle {rt(t-r)\over 2\pi (\alpha t(t-r)+K(t-2r))^2}}} {\mathrm e}^{-\frac{\alpha ^2t(t-r)}{2r}-\frac{\alpha K(t-2r)}{r}-\frac{K^2(t-2r)^2}{2tr(t-r)}}. \end{aligned}$$

Recalling that $g_t=t-h_t$, we get that ${\mathbb {P}}\left( g_t\ge q\right) ={\mathbb {P}}\left( h_t\le t-q\right)$ and hence the assertion of the lemma follows.$\square$

We compute the probability density of the distribution of $g_t$ by differentiating (4.9). This gives the nice formula

4.16 $$\begin{aligned} {\mathbb {P}}\left( g_t\in du\right) =(\alpha t +K)\sqrt{{\textstyle {t\over 2\pi u(t-u)^3}}} {\mathrm e}^{-\frac{t(\alpha u+K)^2}{2u(t-u)}} du. \end{aligned}$$

Thus, by (4.6),

4.17 $$\begin{aligned} {\mathbb {P}}\left( T^K_t>st\right)= & {} \int _{st}^t {\mathbb {P}}\left( g_t\in du\right) \phi _K(u,st)\nonumber \\= & {} \int _{st}^t (\alpha t +K) \sqrt{{\textstyle {t\over 2\pi u(t-u)^3}}} {\mathrm e}^{-\frac{t(\alpha u+K)^2}{2u(t-u)}} \phi _K(u,st)du\nonumber \\= & {} \int _{0}^{1-s} (\alpha +K/t) \sqrt{{\textstyle {1\over 2\pi (s+v)(1-s-v)^3}}} {\mathrm e}^{-\frac{t(\alpha s+v+K)^2}{2(s+v)(1-s-v)}}\nonumber \\{} & {} \quad \times \sqrt{t}\phi _K(st+vt,st) dv, \end{aligned}$$

where we used (4.7) together with (4.16). We use the Laplace method to compute the integral in (4.17). The exponential term takes its maximum at $v=s$. Thus we need to compute the behaviour of the prefactor at *s*. Let us first consider the more complicated case $K>0$. We get

4.18 $$\begin{aligned} \phi _K(st+x,st)= & {} -2\left( {\textstyle {st\over st+x}}\left( 1-{\textstyle {K^2\over st+x}}\right) -1\right) \Phi \left( -{\textstyle {K\sqrt{st}\over \sqrt{(st+x)x}}}\right) -{\textstyle {K\sqrt{2st x}\over \sqrt{\pi (st+x)^3}}}{\mathrm e}^{-\frac{K^2st}{2(st+x)x}}\nonumber \\\sim & {} {\mathrm e}^{-{\textstyle {K^2st\over 2(st+x)x}}}\sqrt{x(st+x)}\left( {\textstyle {2x\over K\sqrt{s}t (st+x)}}+{\textstyle {K \over st^{3/2}}} -{\textstyle {K\over \sqrt{\pi (st+x)^3}}}\right) \nonumber \\\sim & {} {\mathrm e}^{-{\textstyle {K^2\over 2x}}}\sqrt{x}{\textstyle {K \over st}}(1-1/\sqrt{\pi }), \end{aligned}$$

as $$x\downarrow 0$$. Hence,

4.19 $$\begin{aligned} t^{1/2}\phi _K(st+vt,st)=K{\mathrm e}^{-{\textstyle {K^2\over 2vt}}}{\textstyle {\sqrt{v} \over s}}(1-1/\sqrt{\pi })(1+o(1)). \end{aligned}$$

Similarly,

4.20 $$\begin{aligned} \sqrt{{\textstyle {1\over 2\pi (s+v)(1-s-v)^3}}} {\mathrm e}^{-\frac{t(\alpha s+v+K)^2}{2(s+v)(1-s-v)}} \sim \sqrt{{\textstyle {1\over 2\pi s(1-s)^3}}} {\mathrm e}^{-\frac{t\alpha ^2s}{2(1-s)}-\frac{\alpha K}{1-s}}{\mathrm e}^{-vt\frac{\alpha ^2}{2(1-s)^2}}. \end{aligned}$$

Inserting these asymptotics into (4.17), we find that, up to errors of order 1/*t*,

4.21 $$\begin{aligned} {\mathbb {P}}\left( T^K_t>st\right)= & {} \alpha K(1-1/\sqrt{\pi }) \sqrt{{\textstyle {1\over 2\pi s^3(1-s)^3}}} {\mathrm e}^{-\frac{t\alpha ^2s}{2(1-s)}-\frac{\alpha K}{1-s}}\nonumber \\{} & {} \times \int _{0}^{1-s} {\mathrm e}^{-vt\frac{\alpha ^2}{2(1-s)^2}- \frac{K^2}{2vt}}\sqrt{v} dv. \end{aligned}$$

Finally, as $t\uparrow \infty$, substituting $z=vt\frac{\alpha ^2}{2(1-s)^2}$,

4.22 $$\begin{aligned} \int _{0}^{1-s} {\mathrm e}^{-vt\frac{\alpha ^2}{2(1-s)^2}- \frac{K^2}{2vt}}\sqrt{v} dv\sim & {} {\textstyle {2(1-s)^2\over \alpha t^{3/2}}}\int _0^\infty {\mathrm e}^{-z- \frac{K^2\alpha ^2}{2z(1-s)^2}}\sqrt{z} dz\nonumber \\= & {} {\textstyle {(1-s)^2\over \alpha t^{3/2}}}\sqrt{\pi }\left( {\textstyle {\sqrt{2}K\alpha \over 1-s}}+1\right) {\mathrm e}^{-\frac{\sqrt{2} K\alpha }{1-s}}(1+o(1)),\nonumber \\ \end{aligned}$$

so that finally

4.23 $$\begin{aligned} {\mathbb {P}}\left( T^K_t>st\right)= & {} t^{-3/2} K(\sqrt{\pi }-1)\nonumber \\{} & {} \times \sqrt{{\textstyle { 1-s\over 2\pi s^3}}} {\mathrm e}^{-\frac{t\alpha ^2s}{2(1-s)}-\frac{\alpha K(1+\sqrt{2})}{1-s}} \left( {\textstyle {\sqrt{2}K\alpha \over 1-s}}+1\right) (1+o(1)). \end{aligned}$$

In the remaining cases we get

4.24 $$\begin{aligned} \phi _K(ts+tv,ts) ={\left\{ \begin{array}{ll} {\textstyle {v\over s}}, &{}\;\text {if }\; K=0,\\ t^{-1/2}{\textstyle {\sqrt{2v}2|K|\over s\sqrt{\pi }}}, &{}\;\text {if }\; K<0. \end{array}\right. } \end{aligned}$$

Therefore, using Lemma A.2,

4.25 $$\begin{aligned}{} & {} \int _{0}^{1-s} (\alpha +K/t) \sqrt{{\textstyle {1\over 2\pi (s+v)(1-s-v)^3}}} {\mathrm e}^{-\frac{t(\alpha s+v+K)^2}{2(s+v)(1-s-v)}} \sqrt{t}\phi _K(st+vt,st) dv \nonumber \\{} & {} \quad =\left( 1+o(1)\right) \alpha \sqrt{{\textstyle {1\over 2\pi s(1-s)^3}}} {\mathrm e}^{-\frac{t\alpha ^2s}{2(1-s)}-\frac{\alpha K}{1-s}} \int _0^{1-s}dv {\mathrm e}^{-vt\frac{\alpha ^2}{2(1-s)^2}} \nonumber \\{} & {} \qquad \times {\left\{ \begin{array}{ll} {\textstyle {v\over s}},&{}\;\text {if }\; K=0 \\ t^{-1/2}{\textstyle {\sqrt{2v}2|K|\over s\sqrt{\pi }}}, &{}\;\text {if }\; K<0\\ \end{array}\right. } \nonumber \\{} & {} \quad = \left( 1+o(1)\right) t^{-3/2}\alpha \sqrt{{\textstyle {1\over 2\pi s^3(1-s)^3}}} {\mathrm e}^{-\frac{t\alpha ^2s}{2(1-s)}-\frac{\alpha K}{1-s}} \nonumber \\{} & {} \qquad \times {\left\{ \begin{array}{ll} \left( {\textstyle {2(1-s)^2\over \alpha ^2}}\right) ^2,&{}\;\text {if }\; K=0, \\ \left( {\textstyle {2(1-s)^2\over \alpha ^2}}\right) ^{3/2} {\textstyle {\sqrt{2}|K|\over \sqrt{\pi }}},&{}\;\text {if }\; K<0. \end{array}\right. } \end{aligned}$$

(4.25) and (4.23) yield the assertion of Theorem 4.1. $\square$

The control of the distribution of $T^K_t$ given by Theorem 4.1 suffice to prove upper bounds on *w* and hence upper bounds on the wave speed. To prove lower bounds, it is also necessary to take possible fluctuations of the Brownian bridges in the negative direction into account. Therefore, we need on the distribution of $T^K_t$ a lower bound where excursions of the Brownian bridge below zero are suppressed. We define, for $b>0$,

4.26 $$\begin{aligned} U_t^b\equiv \int _0^t \mathbbm {1}_{{{\mathfrak {z}}}^t_{0,0}(s)\le -b}ds. \end{aligned}$$

We want a lower bound on

4.27 $$\begin{aligned} {\mathbb {P}}\left( \{T^K_t>S\} \cap \{ U_t^b\le L\}\right) . \end{aligned}$$

The following lemma is not optimal but sufficient for our purposes.

#### Lemma 1.7

For $$K> 0$$, $$b>0$$, and $$L>0$$,

4.28 $$\begin{aligned}{} & {} {\mathbb {P}}\left( \{T^K_t>st\} \cap \{ U_t^b\le L\}\right) \nonumber \\{} & {} \quad \ge C t^{-3/2}{\mathrm e}^{-{\textstyle {K^2\over 2L}}} \sqrt{L}K \sqrt{{\textstyle {1-s\over 2\pi \alpha ^2}}} {\mathrm e}^{-\frac{t\alpha ^2s}{2(1-s)}-\frac{\alpha ^2L+\alpha K}{1-s}} \left( 1-{\mathrm e}^{-\frac{2b\alpha s}{1-s}}\right) . \end{aligned}$$

#### Proof

Given $g_t$, we use that

4.29 $$\begin{aligned} \left\{ U_t^b\le L\right\} \supseteq \left\{ {{\mathfrak {z}}}^t_{0,0}(s) \ge -b, \forall g_t\le s\le t\right\} \cap \left\{ \int _0^{g_t} \mathbbm {1}_{{{\mathfrak {z}}}_{0,0}^t(s)\le -b} ds\le L\right\} .\nonumber \\ \end{aligned}$$

The second event in turn contains the event that $\{S>g_t-L\}$.

Hence, the main effort is to control the law of $g_t$ under the restriction that the bridge remains above $-b$. By the same reasoning as before, this amounts to proving a lower bound on

4.30 $$\begin{aligned} {\mathbb {P}}\left( \{h_t\le t-u \}\cap \{ {{\mathfrak {z}}}_{0,0}^t(s)\ge -b,\, \forall s\le h_t\}\right) . \end{aligned}$$

To bound this, we have to revisit and alter the proof in Beghin and Orsingher (1999). First, we note that

4.31 $$\begin{aligned}{} & {} {\mathbb {P}}\left( \{h_t\le r\} \cap \{ {{\mathfrak {z}}}_{0,0}^t(s)\ge -b,\, \forall s\le h_t\}\right) \nonumber \\{} & {} \quad ={\mathbb {P}}\left( \left\{ \max _{0\le s\le r} {{\mathfrak {z}}}_{0,0}^t(s)\ge \alpha (t-s) +K\right\} \cap \left\{ \min _{0\le s\le r}{{\mathfrak {z}}}_{0,0}^t(s)\ge -b\right\} \right) . \end{aligned}$$

The latter probability can be written up to normalisation as

4.32 $$\begin{aligned} {\mathbb {P}}\left( \left\{ \max _{0\le s\le r} B(s)\ge \alpha (t-s) +K\right\} \cap \left\{ \min _{0\le s\le r}B(s)\ge -b\right\} \cap \left\{ B(t)=0\right\} \right) ,\nonumber \\ \end{aligned}$$

where *B* is a Brownian motion started in zero. Decomposing this over the values of *B*(*r*) gives

4.33 $$\begin{aligned}{} & {} {\mathbb {P}}\left( \left\{ \max _{0\le s\le r} B(s)\ge \alpha (t-s) +K\right\} \cap \left\{ \min _{0\le s\le r}B(s)\ge -b\right\} \cap \left\{ B(t)=0\right\} \right) \nonumber \\{} & {} \quad =\int _{-b}^\infty {\mathbb {P}}\left( \left\{ \max _{0\le s\le r} B(s)\ge \alpha (t-s) +K\right\} \cap \left\{ \min _{0\le s\le r}B(s)\ge -b\right\} \cap \left\{ B(r)\in dz\right\} \right) \nonumber \\{} & {} \qquad \times \,{\mathbb {P}}\left( B(t)=0\big | B(r)=z\right) .\nonumber \\{} & {} \quad \ge \int _{\alpha (t-r)+K}^\infty {\mathbb {P}}\left( \left\{ \max _{0\le s\le r} B(s)\ge \alpha (t-s) +K\right\} \cap \left\{ \min _{0\le s\le r}B(s)\ge -b\right\} \cap \left\{ B(r)\in dz\right\} \right) \nonumber \\{} & {} \qquad \times \,{\mathbb {P}}\left( B(t)=0\big | B(r)=z\right) \nonumber \\{} & {} \quad \equiv G^>(t-r). \end{aligned}$$

Now, if $z>\alpha (t-r)+K$ then *B*(*r*) is above the line $\alpha (t-s)$ at $s=r$ and a fortiori $\max _{0\le s\le r} B(s)\ge \alpha (t-s) +K$. Hence for these values of *z*,

4.34 $$\begin{aligned}{} & {} {\mathbb {P}}\left( \left\{ \max _{0\le s\le r} B(s)\ge \alpha (t-s) +K\right\} \cap \left\{ \min _{0\le s\le r}B(s)\ge -b\right\} \cap \left\{ B(r)\in dz\right\} \right) \nonumber \\{} & {} \quad ={\mathbb {P}}\left( \left\{ \min _{0\le s\le r}B(s)\ge -b\right\} \cap \left\{ B(r)\in dz\right\} \right) \nonumber \\{} & {} \quad ={\mathbb {P}}\left( B(r)\in dz\right) -{\mathbb {P}}\left( \left\{ \min _{0\le s\le r}B(s)\le -b\right\} \cap \left\{ B(r)\in dz\right\} \right) . \end{aligned}$$

For the last probability we have by the reflection principle that

4.35 $$\begin{aligned} {\mathbb {P}}\left( \left\{ \min _{0\le s\le r}B(s)\le -b\right\} \cap \left\{ B(r)\in dz\right\} \right) ={\mathbb {P}}\left( B(r)\in d(-z-2b)\right) . \end{aligned}$$

The probability in (4.34) is thus given by

4.36 $$\begin{aligned} \frac{1}{\sqrt{2\pi r}} {\mathrm e}^{-\frac{ z^2}{2r}} \left( 1-{\mathrm e}^{-\frac{2bz+2b^2}{r}}\right) dz. \end{aligned}$$

Hence,

4.37 $$\begin{aligned} G^>(t-r)= & {} \frac{1}{2\pi \sqrt{ r(t-r)}} \int _{\alpha (t-r)+K}^\infty {\mathrm e}^{-\frac{ z^2}{2r}} \left( 1-{\mathrm e}^{-\frac{2bz+2b^2}{r}}\right) {\mathrm e}^{-\frac{z^2}{2(t-r)}} dz\nonumber \\= & {} \frac{1}{2\pi \sqrt{ r(t-r)}} \int _{\alpha (t-r)+K}^\infty {\mathrm e}^{-\frac{ z^2t}{2r(t-r)}} \left( 1-{\mathrm e}^{-\frac{2bz+2b^2}{r}}\right) dz. \end{aligned}$$

Passing back to the Brownian bridge, this yields

4.38 $$\begin{aligned} {\mathbb {P}}\left( \{g_t\ge u\} \cap \{ {{\mathfrak {z}}}_{0,0}^t(s)\ge -b,\, \forall s\le h_t\}\right) \ge \sqrt{2\pi t}G^>(u) \end{aligned}$$

Since $\phi _K(g_t, S)$ is monotone increasing in $g_t$, it holds that

4.39 $$\begin{aligned}{} & {} {\mathbb {P}}\left( \{T^K_t>S\} \cap \{ U_t^b\le L\}\right) \nonumber \\{} & {} \quad \ge \int _{S+L}^t {\mathbb {P}}\left( \{g_t\in du\} \cap \left\{ {{\mathfrak {z}}}_{0,0}^t(s)\ge -b,\forall u\le s\le t\right\} \right) \phi _K(u,u-L)\nonumber \\{} & {} \quad \ge \min _{u\in [S+L,t]} \phi _K(u,u-L) \sqrt{2\pi t} G^>(S+L). \end{aligned}$$

Using (4.18), for *L* finite and $S=st$,

4.40 $$\begin{aligned}{} & {} {\mathbb {P}}\left( \{T^K_t>st\} \cap \{U_t^b\le L\}\right) \nonumber \\{} & {} \qquad \sim {\mathrm e}^{-{\textstyle {K^2\over 2L}}}\sqrt{L}{\textstyle {K \over t}}(1-1/\sqrt{\pi }) \sqrt{{\textstyle { t\over {2\pi (st+L) (t-st-L)}}} } \nonumber \\{} & {} \qquad \times \int _{\alpha (st+L)+K}^\infty {\mathrm e}^{-\frac{ z^2t}{2(st+L)(t-st-L)}} \left( 1-{\mathrm e}^{-\frac{2bz+2b^2}{t-st-L}}\right) dz \nonumber \\{} & {} \quad \ge {\mathrm e}^{-{\textstyle {K^2\over 2L}}}\sqrt{L}{\textstyle {K \over t}}(1-1/\sqrt{\pi }) \sqrt{{\textstyle {{(st+L)(t-st-L)}\over {2\pi (\alpha (st+L)+K)^2 t}}}} {\mathrm e}^{-\frac{ t(\alpha ( s+L/t)+K/t)^2}{2(s+L/t)(1-s-L/t)}} \left( 1-{\mathrm e}^{-\frac{2b\alpha s}{1-s}}\right) \nonumber \\{} & {} \quad \ge {\mathrm e}^{-{\textstyle {K^2\over 2L}}}\sqrt{L}{\textstyle {K \over t}}(1-1/\sqrt{\pi }) \sqrt{{\textstyle {(1-s)\over 2\pi \alpha ^2 st }}} {\mathrm e}^{-\frac{t\alpha ^2s}{2(1-s)}-\frac{\alpha ^2L+\alpha K}{1-s}} \left( 1-{\mathrm e}^{-\frac{2b\alpha s}{1-s}}\right) \left( 1+O(1/t)\right) \nonumber \\{} & {} \quad \ge C t^{-3/2}{\mathrm e}^{-{\textstyle {K^2\over 2L}}}\sqrt{L}K \sqrt{{\textstyle {1-s\over 2\pi \alpha ^2}}} {\mathrm e}^{-\frac{t\alpha ^2s}{2(1-s)}-\frac{\alpha ^2L+\alpha K}{1-s}} \left( 1-{\mathrm e}^{-\frac{2b\alpha s}{1-s}}\right) , \end{aligned}$$

for some $$C>0$$. $$\square $$

#### Remark

Note that, up to constants, the difference between the expression for $P(T^K_t>st)$ is that a factor 1/*s* is missing; this is due to the lower bound in (4.39). To keep the difference in upper and lower bound of polynomial order in *t* one could choose $L\sim K$ and

4.41 $$\begin{aligned} |K|\le C\ln (t). \end{aligned}$$

### The Laplace transforms

As seen in (3.14), we need to control the Laplace transform of $T^K_t$. The behaviour of the Laplace transform is very different weither $2\lambda >\alpha ^2$ or $2\lambda \le \alpha ^2$.

#### Lemma 1.8

Assume that $2\lambda >\alpha ^2$. Then, as $t\uparrow \infty$,

$$\begin{aligned} {\mathbb { E}}\left[ {\mathrm e}^{\lambda T^K_t}\right] = {\mathrm e}^{t\frac{(\alpha -\sqrt{2\lambda })^2}{2}-K\sqrt{2\lambda }} {\textstyle {\sqrt{2\alpha }\over \sqrt{\pi (\sqrt{2\lambda }-\alpha )^3}}}\times {\left\{ \begin{array}{ll} \left( 1+o(1)\right) ,&{}\;\text {if }\; K=0, \\ {\sqrt{2\lambda }|K|} \left( 1+o(1)\right) ,&{}\;\text {if }\; K<0, \end{array}\right. } \end{aligned}$$

and, if $$K>0$$,

4.42 $$\begin{aligned} {\mathbb { E}}\left[ {\mathrm e}^{\lambda T^K_t}\right]= & {} {\mathrm e}^{t\frac{(\alpha -\sqrt{2\lambda })^2}{2}-K\sqrt{2\lambda }(1+\sqrt{2})} K(\sqrt{\pi }-1)\nonumber \\{} & {} \times \sqrt{{\textstyle {\alpha \lambda \over 4\pi (\sqrt{2\lambda }-\alpha )^3}}}\left( 2K\sqrt{\lambda }+1\right) (1+o(1)). \end{aligned}$$

#### Proof

Note first that for any non-negative random variable *T*,

4.43 $$\begin{aligned} {\mathbb { E}}\left[ {\mathrm e}^{\lambda T}\right] =\int _0^\infty {\mathbb {P}}\left( T\ge {\textstyle {\ln y\over \lambda }}\right) dy =\int _{-\infty }^\infty \lambda {\mathrm e}^{\lambda r}{\mathbb {P}}\left( T\ge r\right) dr. \end{aligned}$$

From Theorem 4.1 we see that, for $0<r<t$,

4.44 $$\begin{aligned} {\mathbb {P}}\left( T^K_t\ge r\right) =t^{-3/2}P_K(t,r/t) {\mathrm e}^{-\frac{\alpha ^2 tr}{2(t-r)}}, \end{aligned}$$

where $$ P_K(t,s)$$ is polynomially bounded. Moreover, $${\mathbb {P}}\left( T\ge r\right) =1$$ for all $$r\le 0$$ and $${\mathbb {P}}\left( T\ge t\right) =0$$. Thus

4.45 $$\begin{aligned} {\mathbb { E}}\left[ {\mathrm e}^{\lambda T_t}\right]= & {} \int _{-\infty }^0 \lambda {\mathrm e}^{\lambda r}dr +t^{-3/2} \int _{0}^t \lambda {\mathrm e}^{\lambda r} {\mathrm e}^{-\frac{\alpha ^2t r}{2(t-r)}} P_K(t,r/t)(1+o(1))dr \nonumber \\= & {} 1+ t^{-1/2}\int _{0}^1 \lambda {\mathrm e}^{\lambda st} {\mathrm e}^{-t\frac{\alpha ^2 s}{2(1-s)}} P_K(t,s) (1+o(1))ds \end{aligned}$$

Let

4.46 $$\begin{aligned} f(s)=\lambda s-{\textstyle {\alpha ^2s\over 2(1-s)}}, \quad s\in (0,1). \end{aligned}$$

*f*(*s*) takes its maximum in (0, 1) at

4.47 $$\begin{aligned} s^*=1-{\textstyle {\alpha \over \sqrt{2\lambda }}}, \end{aligned}$$

provided that $2\lambda >\alpha ^2$. By an elementary computation,

4.48 $$\begin{aligned} f(s^*)={\textstyle {1\over 2}}\left( \alpha -\sqrt{2\lambda }\right) ^2, \end{aligned}$$

and the second derivative of *f* at $s^*$ is given by

4.49 $$\begin{aligned} f''(s^*) =-{\textstyle {2\lambda \sqrt{2\lambda }\over \alpha }}<0. \end{aligned}$$

Using Lemma A.1 with *f*(*s*) as in (4.46) we get that, if $2\lambda >\alpha ^2$,

4.50 $$\begin{aligned} {\mathbb { E}}\left[ {\mathrm e}^{\lambda T_t^K}\right] =1+\lambda {\textstyle {\sqrt{\alpha }\over (2\lambda )^{3/2}}} {\mathrm e}^{\frac{t\left( \alpha -\sqrt{2\lambda }\right) ^2}{2}} P_K(t,s^*). \end{aligned}$$

where

4.51 $$\begin{aligned} P_K(t,s^*)= & {} {\textstyle {2\lambda \over \sqrt{2\pi \left( 1-\alpha /\sqrt{2\lambda }\right) ^3}}} {\mathrm e}^{ -{ K\sqrt{2\lambda }}} \nonumber \\{} & {} \times {\left\{ \begin{array}{ll} \left( {\textstyle {1\over \lambda }}\right) ^2 {2} \left( 1+o(1)\right) ,&{}\;\text {if }\; K=0, \\ \left( {\textstyle {1\over \lambda }}\right) ^{3/2} \sqrt{2}|K| \left( 1+o(1)\right) ,&{}\;\text {if }\; K<0, \end{array}\right. } \end{aligned}$$

and

4.52 $$\begin{aligned} P_K(t,s^*)= & {} K\left( \sqrt{\pi }-1\right) {\textstyle {2\lambda \over \sqrt{2\pi \left( \sqrt{2\lambda }-\alpha \right) ^3}}} {\mathrm e}^{-\sqrt{2\lambda } K(1+\sqrt{2})} \nonumber \\{} & {} \times \left( {\sqrt{2}K\sqrt{2\lambda }}+1\right) (1+o(1)), \end{aligned}$$

if $K>0$. The claim of Lemma 4.4 follows. $\square$

For the lower bound on *w*, we need to take negative excursions into account, as already mentioned in Sect. 4.1. The following Lemma provides a corresponding bound on the Laplace transform.

#### Lemma 1.9

Assume that $2\lambda >\alpha ^2$ and let $K>0$. Then, as $t\uparrow \infty$,

4.53 $$\begin{aligned} {\mathbb { E}}\left[ {\mathrm e}^{\lambda T_t^K}\mathbbm {1}_{U_t^b\le L}\right]\ge & {} C {\mathrm e}^{-{\textstyle {K^2\over 2L}}}\sqrt{L}K \sqrt{{\textstyle {1\over \left( \sqrt{2\lambda }-\alpha \right) 2\pi \alpha }}}{\mathrm e}^{t\frac{(\alpha -\sqrt{2\lambda })^2}{2}-K\sqrt{2\lambda }(1+\sqrt{2})}\nonumber \\{} & {} \times \left( 1-{\mathrm e}^{-2b\left( \sqrt{2\lambda }-\alpha \right) }\right) . \end{aligned}$$

#### Proof

The proof is a rerun of the proof of Lemma 4.4 using Lemma 4.3 instead of Theorem 4.1. $\square$

Finally, we need bounds on the Laplace transform when $2\lambda \le \alpha ^2$. The following lemma confirms that in this case the Laplace transform is essentially of order one.

#### Lemma 1.10

If $$2\lambda \le \alpha ^2$$ and $$K<0$$,

4.54 $$\begin{aligned} 1\le {\mathbb { E}}\left[ {\mathrm e}^{\lambda T^K_t}\right] \le 1+ {\left\{ \begin{array}{ll} \frac{2}{\sqrt{\pi K}} \lambda {\mathrm e}^{-2\lambda K/\alpha }\frac{2}{\alpha ^2-2\lambda }, &{} \text{ if } 2\lambda <\alpha ^2\\ \frac{2}{\sqrt{\pi K}} \lambda {\mathrm e}^{-2\lambda K/\alpha } (t+2K/\alpha ), &{} \text{ if } 2\lambda =\alpha ^2 \end{array}\right. }. \end{aligned}$$

and

4.55 $$\begin{aligned} {\mathbb { E}}\left[ {\mathrm e}^{\lambda T_t^K}\mathbbm {1}_{U_t^b\le L}\right] \ge 1-{\mathrm e}^{-b^2/2t}. \end{aligned}$$

#### Proof

Starting from (4.43) we simply use

4.56 $$\begin{aligned} {\mathbb {P}}(T_t^K\ge r)\le {\mathbb {P}}(g_t\ge r). \end{aligned}$$

The latter has been computed in Lemma 4.2 and we have, for $\alpha r>-2K$,

4.57 $$\begin{aligned} {\mathbb {P}}(g_t\ge r)= & {} {\mathrm e}^{-\frac{2K(\alpha t+K)}{t}} \Phi \left( -{\textstyle {(\alpha tr+K(2r-t))\over \sqrt{rt(t-r)}}}\right) +1- \Phi \left( {\textstyle {(\alpha tr)+Kt)\over \sqrt{rt(t-r)}}}\right) \end{aligned}$$

4.58 $$\begin{aligned}\le & {} {\mathrm e}^{-2K\alpha } \frac{\sqrt{rt(t-r)}}{(\alpha tr+K(2r-t))\sqrt{2\pi }}{\mathrm e}^{-\frac{\left( \alpha tr+K(2r-t)\right) ^2}{2rt(t-r)}} +\frac{\sqrt{rt(t-r)}}{(\alpha tr+Kt)\sqrt{2\pi }}{\mathrm e}^{-\frac{\left( \alpha tr+Kt\right) ^2}{2rt(t-r)}}\nonumber \\\le & {} \frac{\sqrt{rt(t-r)}}{\sqrt{2\pi }} {\mathrm e}^{-\frac{\alpha K t}{t-r} -\frac{\alpha ^2 tr}{2(t-r)}}\left( \frac{1}{\alpha tr+K(2r-t)}+ \frac{1}{\alpha tr+Kt}\right) \nonumber \\\le & {} \frac{4}{\sqrt{2\pi \alpha r}} {\mathrm e}^{-\frac{\alpha K t}{t-r}-\frac{\alpha ^2 tr}{2(t-r)}}, \end{aligned}$$

by standard Gaussian tail bounds. Plugging this into (4.43) we get

4.59 $$\begin{aligned} {\mathbb { E}}\left[ {\mathrm e}^{\lambda T^K_t}\right]&\le \int _{-\infty }^{-2K/\alpha } \lambda {\mathrm e}^{\lambda r} dr + \int _{-2K/\alpha }^t \lambda {\mathrm e}^{\lambda r}\frac{4}{\sqrt{2\pi \alpha r}} {\mathrm e}^{-\frac{\alpha K t}{t-r}-\frac{\alpha ^2 tr}{2(t-r)}}dr\nonumber \\&= e^{-2K\lambda /\alpha } + \int _{0}^{t+2K/\alpha } \lambda {\mathrm e}^{\lambda z-\lambda 2K/\alpha }\frac{4}{\sqrt{2\pi \alpha (z-2K/\alpha )}} {\mathrm e}^{ -\frac{\alpha ^2 tz}{2(t-z+2K/\alpha )}}dz, \end{aligned}$$

where $z=r+2K/\alpha$. The second summand in (4.59) is bounded from above by

4.60 $$\begin{aligned}{} & {} \frac{2}{\sqrt{\pi K}} \lambda {\mathrm e}^{-2\lambda K/\alpha } \int _{0}^{t+2K/\alpha } {\mathrm e}^{\lambda z -\frac{\alpha ^2 z}{2}}dz \nonumber \\{} & {} \quad = {\left\{ \begin{array}{ll} \frac{2}{\sqrt{\pi K}} \lambda {\mathrm e}^{-2\lambda K/\alpha }\frac{2}{\alpha ^2-2\lambda }, &{} \text{ if } 2\lambda <\alpha ^2\\ \frac{2}{\sqrt{\pi K}} \lambda {\mathrm e}^{-2\lambda K/\alpha } (t+2K/\alpha ), &{} \text{ if } 2\lambda =\alpha ^2 \end{array}\right. }. \end{aligned}$$

To prove (4.55) we bound the left hand side of (4.55) from below by

4.61 $$\begin{aligned} {\mathbb {P}}\left( U_t^b\le L\right) \ge {\mathbb {P}}\left( U_t^b=0\right) = 1-{\mathrm e}^{-b^2/2t}. \end{aligned}$$

This finishes the proof of Lemma 4.6. $\square$

## Controlling the wave

We use the Brownian bridge estimates from the previous section to give a rigorous version of the heuristics outlined at the end of Sect. 3.

### Bounds on the speed of the wave

We first control the behaviour of solutions on the exponential scale for large *t*. We begin with an upper bound.

#### Lemma 1.11

(i) Let *u* be such that
  5.1 $$\begin{aligned} 2\left( 1-{\tilde{\gamma }}\right) > \left( \sqrt{2}-u\right) ^2, \end{aligned}$$
  Then, for all $\epsilon >0$ small enough, there exists a constant $C>0$ such that
  5.2 $$\begin{aligned} w(t,ut)\le \frac{C}{u\sqrt{t}}{\mathrm e}^{t \left( 2-{\tilde{\beta }}-2\sqrt{(1-{\tilde{\gamma }})(1-\epsilon )}+\sqrt{2} u\left( \sqrt{(1-{\tilde{\gamma }})(1-\epsilon )}-1\right) \right) }. \end{aligned}$$
  In particular, *w*(*t*, *ut*) decays exponentially fast in *t* for
  5.3 $$\begin{aligned} u>u^*\equiv \sqrt{2}-\frac{{\tilde{\beta }}\left( \sqrt{1-{\tilde{\gamma }}}+1\right) }{\sqrt{2} {\tilde{\gamma }}}. \end{aligned}$$
(ii) Let *u* be such that
  5.4 $$\begin{aligned} 2\left( 1-{\tilde{\gamma }}\right) \le \left( \sqrt{2}-u\right) ^2, \end{aligned}$$
  Then, for all $\epsilon >0$ small enough, there exists a constant $C>0$ such that
  5.5 $$\begin{aligned} w(t,ut)\le \frac{C}{u\sqrt{t}}{\mathrm e}^{-{\textstyle {u^2t\over 2}}+t( ({\tilde{\gamma }}-{\tilde{\beta }}) +\epsilon (1-{\tilde{\gamma }})) } \end{aligned}$$

#### Remark

Lemma 5.1 implies that the solution is exponentially small if $u>u_c$ [given in (2.17)], hence the wave speed is not larger than $u_c$.

#### Proof

We bound the integral in the Feynman–Kac representation (3.3) from above as follows.

5.6 $$\begin{aligned}{} & {} \int _0^t \biggl (1-{\tilde{\beta }}-(1-{\tilde{\gamma }})\omega \left( x{\textstyle {t-s\over t}} +{\textstyle {s\over t}} y +{{\mathfrak {z}}}^t_{0,0}(s)+a-\sqrt{2}(t-s)\right) \nonumber \\{} & {} \qquad -\,{\tilde{\gamma }}w\left( t-s, x{\textstyle {t-s\over t}} +{\textstyle {s\over t}} y +{{\mathfrak {z}}}_{0,0}^t(s)\right) \biggr )ds\nonumber \\{} & {} \quad \le \int _0^t \left( \mathbbm {1}_{{{\mathfrak {z}}}_{0,0}^t(s)\ge (\sqrt{2}-x/t)(t-s) +K} +\mathbbm {1}_{{{\mathfrak {z}}}_{0,0}^t(s)< (\sqrt{2}-x/t)(t-s) +K}\right) \nonumber \\{} & {} \qquad \times \,\left( 1-{\tilde{\beta }}-(1-{\tilde{\gamma }})\omega \left( x{\textstyle {t-s\over t}} +{\textstyle {s\over t}} y +{{\mathfrak {z}}}^t_{0,0}(s)+a-\sqrt{2}(t-s)\right) \right) ds.\qquad \end{aligned}$$

Using the asymptotic of the lower tail (2.13), we see that on the second indicator function, for all $y\le 0$,

5.7 $$\begin{aligned} \omega \left( x{\textstyle {t-s\over t}} +{\textstyle {s\over t}} y +{{\mathfrak {z}}}^t_{0,0}(s)+a-\sqrt{2}(t-s)\right) \ge \omega \left( a+K \right) \ge 1-c {\mathrm e}^{(2-\sqrt{2})(a+K)},\nonumber \\ \end{aligned}$$

which is larger than $1-\epsilon$ for $-K$ large enough. On the first indicator function we use that $\omega \ge 0$. This leads to

5.8 $$\begin{aligned}{} & {} \int _0^t 1-{\tilde{\beta }}-(1-{\tilde{\gamma }})\omega \left( x{\textstyle {t-s\over t}} +{\textstyle {s\over t}} y +{{\mathfrak {z}}}^t_{0,0}(s)+a-\sqrt{2}(t-s)\right) ds\nonumber \\{} & {} \quad \le ({\tilde{\gamma }}-{\tilde{\beta }})t+\epsilon (1-{\tilde{\gamma }})t+(1-{\tilde{\gamma }})(1-\epsilon )T^{K}_t. \end{aligned}$$

Recalling (3.7), if $2\left( 1-{\tilde{\gamma }}\right) > \left( \sqrt{2}-u\right) ^2$, we obtain the upper bound

5.9 $$\begin{aligned} w(t,x)\le & {} {\textstyle {1-{\tilde{\beta }}/{\tilde{\gamma }}\over \sqrt{2\pi t}}}\int _{-\infty }^0 {\mathrm e}^{-{\textstyle {(x-y)^2\over 2t}}} {\mathrm e}^{t( ({\tilde{\gamma }}-{\tilde{\beta }}) +\epsilon (1-{\tilde{\gamma }}) )} {\mathbb { E}}\left[ {\mathrm e}^{(1-{\tilde{\gamma }})(1-\epsilon )T^K_t}\right] dy \nonumber \\\le & {} {\textstyle {1-{\tilde{\beta }}/{\tilde{\gamma }}\over \sqrt{2\pi x^2/t}}} {\mathrm e}^{-{\textstyle {x^2\over 2t}}} {\mathrm e}^{t( ({\tilde{\gamma }}-{\tilde{\beta }}) +\epsilon (1-{\tilde{\gamma }})) } {\mathrm e}^{t{\left( (\sqrt{2}-x/t)-\sqrt{2(1-{\tilde{\gamma }})(1-\epsilon )}\right) ^2}/2-K\sqrt{2(1-{\tilde{\gamma }})(1-\epsilon )}} \nonumber \\{} & {} \times {\textstyle {\sqrt{2(\sqrt{2}-x/t)}\over \sqrt{\pi (\sqrt{2(1-{\tilde{\gamma }})(1-\epsilon )}-(\sqrt{2}-x/t))^3}}} {\sqrt{2(1-{\tilde{\gamma }})}|K|} \left( 1+o(1)\right) . \end{aligned}$$

The exponential terms are (for $x=ut$),

5.10 $$\begin{aligned}{} & {} {\mathrm e}^{-t\left( {\textstyle {u^2\over 2}}+ {\tilde{\beta }}-{\tilde{\gamma }}-\epsilon (1-{\tilde{\gamma }}) -{\left( (\sqrt{2}-u)-\sqrt{2(1-{\tilde{\gamma }})(1-\epsilon )}\right) ^2}/2\right) } \nonumber \\{} & {} \quad = {\mathrm e}^{t \left( 2-{\tilde{\beta }}-2\sqrt{(1-{\tilde{\gamma }})(1-\epsilon )}+\sqrt{2} u\left( \sqrt{(1-{\tilde{\gamma }})(1-\epsilon )}-1\right) \right) }. \end{aligned}$$

This implies the first part of the Lemma.

For the second one, if $2\left( 1-{\tilde{\gamma }}\right) \le \left( \sqrt{2}-u\right) ^2$ we use the bound from Lemma 4.6 and get

5.11 $$\begin{aligned} w(t,x)\le & {} {\textstyle {1-{\tilde{\beta }}/{\tilde{\gamma }}\over \sqrt{2\pi t}}}\int _{-\infty }^0 {\mathrm e}^{-{\textstyle {(x-y)^2\over 2t}}} {\mathrm e}^{t( ({\tilde{\gamma }}-{\tilde{\beta }}) +\epsilon (1-{\tilde{\gamma }}) )} {\mathbb { E}}\left[ {\mathrm e}^{(1-{\tilde{\gamma }})(1-\epsilon )T^K_t}\right] dy \nonumber \\\le & {} {\textstyle {1-{\tilde{\beta }}/{\tilde{\gamma }}\over \sqrt{2\pi x^2/t}}} {\mathrm e}^{-{\textstyle {x^2\over 2t}}} {\mathrm e}^{t( ({\tilde{\gamma }}-{\tilde{\beta }}) +\epsilon (1-{\tilde{\gamma }})) }\nonumber \\{} & {} \times {\left\{ \begin{array}{ll} 1+ \frac{2(1-{\tilde{\gamma }})}{\sqrt{\pi K}} {\mathrm e}^{-2(1-{\tilde{\gamma }})(1-\epsilon ) K/\alpha }\frac{2}{\alpha ^2-2(1-{\tilde{\gamma }})(1-\epsilon )}, &{} \text{ if } 2(1-{\tilde{\gamma }})(1-\epsilon )<\alpha ^2,\\ 1+ \frac{2(1-{\tilde{\gamma }})}{\sqrt{\pi K}} {\mathrm e}^{-2(1-{\tilde{\gamma }})(1-\epsilon ) K/\alpha } (t+2K/\alpha ), &{} \text{ if } 2(1-{\tilde{\gamma }})(1-\epsilon )=\alpha ^2. \end{array}\right. }\nonumber \\ \end{aligned}$$

The exponential terms are, for $x=ut$,

5.12 $$\begin{aligned} {\mathrm e}^{-{\textstyle {u^2t\over 2}}+t( ({\tilde{\gamma }}-{\tilde{\beta }}) +\epsilon (1-{\tilde{\gamma }})) } \end{aligned}$$

This implies that *w* decays exponentially fast for $u>\sqrt{2({\tilde{\gamma }}-{\tilde{\beta }})}$. $\square$

Next, we need a corresponding lower bound. For this we use the lower bound from Lemma 4.5.

#### Lemma 1.12

Let $b>0$. Assume that *u* is such that $w(t,ut+z)\le \epsilon$, for all $z\ge -2b$ and *t* large enough.

(i) If $2\left( 1-{\tilde{\gamma }}\right) \left( 1-\epsilon \right) > \left( \sqrt{2}-u\right) ^2$ then, for some constant $C>0$ depending on *K*, *L*, *b* and *u*,
  5.13 $$\begin{aligned} w(t,ut) \ge {C} t^{-1/2}{\mathrm e}^{-tu\left( \sqrt{2}-\sqrt{2(1-{\tilde{\gamma }})(1- \epsilon )}\right) }{\mathrm e}^{t\left( 1-\sqrt{(1-{\tilde{\gamma }})(1- \epsilon )}\right) ^2t} {\mathrm e}^{({\tilde{\gamma }}(1-\epsilon )-{\tilde{\beta }}) t}. \end{aligned}$$
  This contradicts the hypothesis, unless
  5.14 $$\begin{aligned} u> \sqrt{2}-{\textstyle {\left( {\tilde{\beta }}+\epsilon \right) \left( \sqrt{\left( 1-{\tilde{\gamma }}\right) \left( 1-\epsilon \right) }+1\right) \over \sqrt{2}\left( {\tilde{\gamma }}(1-\epsilon ) +\epsilon \right) }}. \end{aligned}$$
(ii) If $2\left( 1-{\tilde{\gamma }}\right) \left( 1-\epsilon \right) \le \left( \sqrt{2}-u\right) ^2$, then there exists a constant $C>0$ depending on *K*, *b* and *u*
  5.15 $$\begin{aligned} w(t,ut)\ge \frac{C}{t^{3/2}} {\mathrm e}^{({\tilde{\gamma }}(1-\epsilon )-{\tilde{\beta }}) t-\frac{u^2t}{2}} \end{aligned}$$
  This contradicts the hypothesis unless
  5.16 $$\begin{aligned} u>\sqrt{2\left( {\tilde{\gamma }}(1-\epsilon )-{\tilde{\beta }}\right) }. \end{aligned}$$

#### Remark

From (5.14) it follows that the speed is not smaller than $\sqrt{2}{-}\frac{{\tilde{\beta }}}{\sqrt{2}{\tilde{\gamma }}} \left( 1{+}\sqrt{1{-}{\tilde{\gamma }}}\right)$ and from (5.16) it follows that it is not smaller than $\sqrt{2\left( {\tilde{\gamma }}-{\tilde{\beta }}\right) }$. Altogether, this implies that the speed is not smaller than the maximum of the two, i.e. it is at least

#### Proof

In the representation (3.3) of *w*(*t*, *x*) we would like to use the assumption of the lemma to argue that the term involving *w* in the exponent is negligible but this could be spoiled by large negative excursions of the Brownian bridge. To avoid this problem we restrict the expectation in (3.3) on the Brownian bridge to a subset $\{U_t^b\le L\}$, For any $L>0$ and $b>0$,

5.17 $$\begin{aligned} w(t,x)\ge & {} {\textstyle {1\over \sqrt{2 \pi t}}}\int _{-b}^0dy {\mathrm e}^{-\frac{(x-y)^2}{2t}} \nonumber \\{} & {} \times \, {\mathbb { E}}\Biggl [\exp \Biggl (\int _0^t \biggl (1-{\tilde{\beta }}-(1-{\tilde{\gamma }})\omega \left( x{\textstyle {t-s\over t}} +{\textstyle {s\over t}} y +{{\mathfrak {z}}}^t_{0,0}(s)+a-\sqrt{2}(t-s)\right) \nonumber \\{} & {} -\,{\tilde{\gamma }}w\left( t-s, x{\textstyle {t-s\over t}} +{\textstyle {s\over t}} y +{{\mathfrak {z}}}_{0,0}^t(s)\right) \biggr )ds\Biggr )\mathbbm {1}_{U_t^b\le L}\Biggr ], \end{aligned}$$

Note that on the event $\{U_t^b\le L\}$, we have

5.18 $$\begin{aligned} -\int _0^t {\tilde{\gamma }}w\left( t-s, x{\textstyle {t-s\over t}} +{\textstyle {s\over t}} y +{{\mathfrak {z}}}_{0,0}^t(s)\right) ds \ge -{\tilde{\gamma }}L-{\tilde{\gamma }}\epsilon t. \end{aligned}$$

Hence, (5.17) is bounded from below by

5.19 $$\begin{aligned}{} & {} {\mathrm e}^{-{\tilde{\gamma }}L+(1-{\tilde{\beta }}-{\tilde{\gamma }}\epsilon ) t}{\textstyle {1-{\tilde{\beta }}/{\tilde{\gamma }}\over \sqrt{2 \pi t}}}\int _{-b}^0dy {\mathrm e}^{-\frac{(x-y)^2}{2t}} \nonumber \\{} & {} \quad \times \, {\mathbb { E}}\Biggl [\exp \Biggl (-\int _0^t (1-{\tilde{\gamma }})\omega \left( x{\textstyle {t-s\over t}} +{\textstyle {s\over t}} y +{{\mathfrak {z}}}^t_{0,0}(s)\right. \nonumber \\{} & {} \quad \left. +\,a-\sqrt{2}(t-s)\right) ds\Biggr )\mathbbm {1}_{U_t^b\le L}\Biggr ]. \end{aligned}$$

The idea is to split the integral in the exponent of the Feynman–Kac formula (3.3) according to the position of the Brownian bridge with respect to the $\omega$-wave, i.e. we write, with $x=ut+z$ and *u* as in the lemma,

5.20 $$\begin{aligned}{} & {} -\int _0^t (1-{\tilde{\gamma }})\omega \left( x{\textstyle {t-s\over t}} +{\textstyle {s\over t}} y +{{\mathfrak {z}}}^t_{0,0}(s)+a-\sqrt{2}(t-s)\right) ds\nonumber \\{} & {} \quad = -\int _0^t \left( \mathbbm {1}_{{{\mathfrak {z}}}_{0,0}^t(s)\ge (\sqrt{2}-x/t)(t-s) +K} +\mathbbm {1}_{{{\mathfrak {z}}}_{0,0}^t(s)< (\sqrt{2}-x/t)(t-s) +K}\right) \nonumber \\{} & {} \qquad \times \, (1-{\tilde{\gamma }})\omega \left( x{\textstyle {t-s\over t}} +{\textstyle {s\over t}} y +{{\mathfrak {z}}}^t_{0,0}(s)+a-\sqrt{2}(t-s)\right) ds. \end{aligned}$$

On the first indicator function we use that

5.21 $$\begin{aligned} \omega \left( x{\textstyle {t-s\over t}} +{\textstyle {s\over t}} y +{{\mathfrak {z}}}^t_{0,0}(s)+a-\sqrt{2}(t-s)\right)\le & {} \omega \left( K+{\textstyle {s\over t}} y +a \right) \nonumber \\\le & {} C (K +a){\mathrm e}^{-\sqrt{2}(K+a)/2}, \end{aligned}$$

if $y\ge - (K+a)/2$. Choosing $K+a$ large enough, we can make this smaller than $\epsilon$, for any $\epsilon >0$. On the second indicator function, we just use that $\omega \le 1$. Thus (5.20) is bounded from below by

5.22 $$\begin{aligned} -(1-{\tilde{\gamma }})\epsilon T_t^K-(1-{\tilde{\gamma }})(t-T_t^K) =-(1-{\tilde{\gamma }})t-(1-{\tilde{\gamma }})(1-\epsilon )T_t^K.\qquad \quad \end{aligned}$$

Inserting this bound into (3.3), we get that

5.23 $$\begin{aligned} w(t,x)&\ge {\mathrm e}^{-{\tilde{\gamma }}L+({\tilde{\gamma }}(1-\epsilon )-{\tilde{\beta }}) t}{\textstyle {1-{\tilde{\beta }}/{\tilde{\gamma }}\over \sqrt{2\pi t}}}\nonumber \\&\times \int _{-\left( (K+a)/2 \wedge b\right) }^0 {\mathrm e}^{-\frac{(x-y)^2}{2t}} {\mathbb { E}}\left[ {\mathrm e}^{(1-{\tilde{\gamma }})(1- \epsilon )T^K_t}\mathbbm {1}_{U_t^b\le L}\right] dy. \end{aligned}$$

*Case 1* $$2\left( 1-{\tilde{\gamma }}\right) \left( 1-\epsilon \right) > \left( \sqrt{2}-u\right) ^2$$.

We insert the lower bound from Lemma 4.5 into (5.23). This gives, if $K>0$,

5.24 $$\begin{aligned}{} & {} w(t,x)\ge {\mathrm e}^{-{\tilde{\gamma }}L+({\tilde{\gamma }}(1-\epsilon )-{\tilde{\beta }}) t}{\textstyle {1-{\tilde{\beta }}/{\tilde{\gamma }}\over \sqrt{2\pi t}}}\nonumber \\{} & {} \quad \int _{-\left( (K+a)/2 \wedge b\right) }^0 {\mathrm e}^{-\frac{(x-y)^2}{2t}}dy C {\mathrm e}^{-{\textstyle {K^2\over 2L}}}\sqrt{L}K \sqrt{{\textstyle {1\over \left( \sqrt{2(1-{\tilde{\gamma }})(1- \epsilon )}-\alpha \right) 2\pi \alpha }}}\nonumber \\{} & {} \qquad \times \, {\mathrm e}^{t\frac{\left( \alpha -\sqrt{2(1-{\tilde{\gamma }})(1- \epsilon )}\right) ^2}{2}-K\sqrt{2(1-{\tilde{\gamma }})(1- \epsilon )}(1+\sqrt{2})}\left( 1-{\mathrm e}^{-2b\left( \sqrt{2(1-{\tilde{\gamma }})(1- \epsilon )}-\alpha \right) }\right) \nonumber \\{} & {} \quad \ge {\mathrm e}^{-\frac{x^2}{2t}}\frac{1}{u}\left( 1-{\mathrm e}^{-\left( (K+a)/2 \wedge b\right) u }\right) {\mathrm e}^{-{\tilde{\gamma }}L+({\tilde{\gamma }}(1-\epsilon )-{\tilde{\beta }}) t}{\textstyle {1-{\tilde{\beta }}/{\tilde{\gamma }}\over \sqrt{2\pi t}}} C {\mathrm e}^{-{\textstyle {K^2\over 2L}}}\nonumber \\{} & {} \qquad \times \sqrt{L}K \sqrt{{\textstyle {1\over \left( \sqrt{2(1-{\tilde{\gamma }})(1- \epsilon )}-\alpha \right) 2\pi \alpha }}}\nonumber \\{} & {} \qquad \times \,{\mathrm e}^{t\frac{\left( \alpha -\sqrt{2(1-{\tilde{\gamma }})(1- \epsilon )}\right) ^2}{2}-K\sqrt{2(1-{\tilde{\gamma }})(1- \epsilon )}(1+\sqrt{2})}\left( 1-{\mathrm e}^{-2b\left( \sqrt{2(1-{\tilde{\gamma }})(1- \epsilon )}-\alpha \right) }\right) ,\nonumber \\ \end{aligned}$$

for $x=ut$. The exponential terms are, for $x=ut$,

5.25 $$\begin{aligned}{} & {} {\mathrm e}^{-t \left( \frac{u^2}{2} -{\tilde{\gamma }}(1-\epsilon ) +{\tilde{\beta }}-\frac{(\sqrt{2}-u-\sqrt{2(1-{\tilde{\gamma }})(1-\epsilon )})^2}{2}\right) } \nonumber \\{} & {} \quad = {\mathrm e}^{t \left( 2-\epsilon -{\tilde{\beta }}-2\sqrt{(1-{\tilde{\gamma }})(1-\epsilon )}+\sqrt{2} u\left( \sqrt{(1-{\tilde{\gamma }})(1-\epsilon )}-1\right) \right) }. \end{aligned}$$

The exponent vanishes if

5.26 $$\begin{aligned} u=u^*\equiv \sqrt{2}-{\textstyle {({\tilde{\beta }}+\epsilon )\left( \sqrt{(1-{\tilde{\gamma }})(1-\epsilon )}+1\right) \over \sqrt{2} (\gamma +\epsilon (1-{\tilde{\gamma }}))}} =\sqrt{2}-{\textstyle {{\tilde{\beta }}\left( \sqrt{(1-{\tilde{\gamma }})}+1\right) \over \sqrt{2} \gamma }} +O(\epsilon ). \end{aligned}$$

and is decreasing in *u*. Hence, for $u<u^*$, this contradicts the hypothesis that $w(t,ut+z)\le \epsilon$. Hence $u^*$ is a lower bound on the wave speed. This concludes the proof of the first part of Lemma 5.2.

*Case 2* $$2\left( 1-{\tilde{\gamma }}\right) \left( 1-\epsilon \right) \le \left( \sqrt{2}-u\right) ^2$$.

In this case we insert the lower bound from Lemma 4.6 into (5.23) and set $L=0$. Hence,

5.27 $$\begin{aligned} w(t,ut)\ge & {} {\mathrm e}^{({\tilde{\gamma }}(1-\epsilon )-{\tilde{\beta }}) t}{\textstyle {1-{\tilde{\beta }}/{\tilde{\gamma }}\over \sqrt{2\pi t}}}\int _{-\left( (K+a)/2 \wedge b\right) }^0 {\mathrm e}^{-\frac{(ut-y)^2}{2t}} \left( 1-{\mathrm e}^{-\frac{b^2}{2t}}\right) dy\nonumber \\\ge & {} {\textstyle {b^2\over 2t}} {\mathrm e}^{({\tilde{\gamma }}(1-\epsilon )-{\tilde{\beta }}) t}{\textstyle {1-{\tilde{\beta }}/{\tilde{\gamma }}\over \sqrt{2\pi t}}}{\mathrm e}^{-\frac{u^2t}{2}}\frac{1}{u}\left( 1-{\mathrm e}^{-\left( (K+a)/2 \wedge b\right) u }\right) , \end{aligned}$$

which implies (5.15). Note that on the exponential scale (5.27) is

5.28 $$\begin{aligned} {\mathrm e}^{-\frac{u^2t}{2}+({\tilde{\gamma }}(1-\epsilon )-{\tilde{\beta }}) t}, \end{aligned}$$

implying (5.16). This finishes the proof of Lemma 5.2. $\square$

### Precise control at the tip of the wave

The estimates obtained on *w*(*t*, *x*) allow for a finer control of the position of the wave as a function of *t*. We assume now that *a* is a constant. The most serious error term in the bounds comes from the $t O(\epsilon )$ in the exponents. To obtain an error of order 1, we want to choose $\epsilon =O(1/t)$ in Lemmas 5.1 and 5.2. To this end, one needs to choose *K* large enough, such that the terms on the right hand side of (5.7) and (5.21) are of order 1/*t*. This requires to choose $K\sim c\ln (t)$. We state precise estimates only for the more interesting case $u^*>\sqrt{2\left( {\tilde{\gamma }}-{\tilde{\beta }}\right) }$, analogous results in the other cases can be obtained in the same way.

#### Lemma 1.13

Assume that ${\tilde{\beta }}$ and ${\tilde{\gamma }}$ are such that $u^*>\sqrt{2\left( {\tilde{\gamma }}-{\tilde{\beta }}\right) }$. Let *a* be a constant independent of *t*. Let $c_+=1/(2-\sqrt{2})$. Then, there exists a constant $0<C<\infty$, independent of *t*, such that, for $x=u^*+z$,

5.29 $$\begin{aligned} w(t,u^*t+z) \le C\frac{1}{u^* }t^{ c_+\sqrt{1-{\tilde{\gamma }}}-1/2}|\ln t| {\mathrm e}^{-z\sqrt{2}\left( 1-\sqrt{1-{\tilde{\gamma }}}\right) }. \end{aligned}$$

In particular, $w(t,u^*t+z)\le C/t$ if, for some ${\delta }>0$,

5.30 $$\begin{aligned} z\ge z_+\equiv {\textstyle {c_+\sqrt{1-{\tilde{\gamma }}}+1/2+{\delta }\over \sqrt{ 2}\left( 1-\sqrt{1-{\tilde{\gamma }}}\right) }} {\ln t}. \end{aligned}$$

#### Proof

The proof is straightforward from (5.9), choosing $K=-c_+\ln t$. $\square$

The next lemma shows that this upper bound is not too bad.

#### Lemma 1.14

Assume that ${\tilde{\beta }}$ and ${\tilde{\gamma }}$ are such that $u^*>\sqrt{2\left( {\tilde{\gamma }}-{\tilde{\beta }}\right) }$. Let *a* be a constant independent of *t*. Then, with $c_-=\sqrt{2}$, there exists a constant $0<C<\infty$, independent of *t*, such that, for $x=u^*t+z$, $w(t,u^*t+z)\le C/t$, then

5.31 $$\begin{aligned} w(t,u^*t+z) \ge C\frac{1}{u^* }t^{- c_- \sqrt{2(1-{\tilde{\gamma }})}(1+\sqrt{2})-1/2} |\ln t|^2 {\mathrm e}^{-z\sqrt{2}\left( 1-\sqrt{1-{\tilde{\gamma }}}\right) }. \end{aligned}$$

In particular, this is in contradiction with the assumption if

5.32 $$\begin{aligned} z\le z_-\equiv -{\textstyle {c_-\sqrt{2(1-{\tilde{\gamma }})}(1+\sqrt{2})-1/2\over \sqrt{ 2}(1-\sqrt{1-{\tilde{\gamma }}})}}\ln t. \end{aligned}$$

#### Proof

This is straightforward from (5.21), choosing $K=c_-\ln t$.$\square$

Lemma 5.4 tells us that $w(t, u^* t +z_-)$ is greater than *O*(1/*t*). The next lemma tells us that at time of order $\ln t$ later, this will have grown to *O*(1).

#### Lemma 1.15

Let $\epsilon (t)>0$. Assume that

5.33 $$\begin{aligned} w(t,ut+z)\ge \epsilon (t) \quad \forall z\le 0. \end{aligned}$$

Then, for all ${\delta }>0$ sufficiently small, there exists a constant *c* such that

5.34 $$\begin{aligned} w\left( t+c\ln \left( \epsilon (t)^{-1}\right) ,ut+z\right) \ge {\delta }\left( 1-\frac{{\tilde{\beta }}}{{\tilde{\gamma }}}\right) >0. \end{aligned}$$

#### Proof

Starting from the Feynman–Kac formula, we have

5.35 $$\begin{aligned} w(t+s,x)= & {} {\mathbb { E}}_x\left[ {\mathrm e}^{\int _0^s \left( 1-{\tilde{\beta }}-(1-{\tilde{\gamma }}) \omega \left( B_r-\sqrt{2}(s-r)\right) -{\tilde{\gamma }}w\left( t+s-r,B_r\right) \right) dr}w\left( t,B_s\right) \right] \nonumber \\= & {} \int _{-\infty }^{\infty } \frac{{\mathrm e}^{-\frac{(x-y)^2}{2s}}}{\sqrt{2\pi s}}\nonumber \\{} & {} \times {\mathbb { E}}\left[ {\mathrm e}^{\int _0^s \left( 1-{\tilde{\beta }}-(1-{\tilde{\gamma }}) \omega \left( x+{{\mathfrak {z}}}^s_{0,x-y}(r)-\sqrt{2}(s-r)\right) -{\tilde{\gamma }}w\left( t+s-r,x+{{\mathfrak {z}}}^s_{0,x-y}(r)\right) \right) dr}w(t,y)\right] \nonumber \\\ge & {} \epsilon (t) \int _{ut-1}^{ut} \frac{{\mathrm e}^{-\frac{(x-y)^2}{2s}}}{\sqrt{2\pi s}} {\mathbb { E}}\left[ {\mathrm e}^{({\tilde{\gamma }}-{\tilde{\beta }})s-{\tilde{\gamma }}\int _0^s w\left( t+s-r,x+\frac{y-x}{s}r+{{\mathfrak {z}}}_{0,0}^s(r)\right) dr}\right] , \end{aligned}$$

where we used that $\omega \le 1$. Plugging in $x=ut+z$ and restricting the Brownian bridge to be larger than $-b$ for some $b>0$, we get that (5.35) is bounded from below by

5.36 $$\begin{aligned} \epsilon (t) \int _{ut-1}^{ut} \frac{{\mathrm e}^{-\frac{(ut+z-y)^2}{2s}}}{\sqrt{2\pi s}} {\mathbb { E}}\left[ {\mathrm e}^{({\tilde{\gamma }}-{\tilde{\beta }})s-{\tilde{\gamma }}\int _0^s w\left( t+s-r,(ut +z)\frac{s-r}{s}+\frac{y}{s}r+{{\mathfrak {z}}}_{0,0}^s(r)\right) dr}\mathbbm {1}_{U_s^b=0} \right] . \end{aligned}$$

Now we assume that for all $0\le r\le s$,

5.37 $$\begin{aligned} w(t+s-r, ut+{{\tilde{z}}})<{\delta }\left( 1-\frac{{\tilde{\beta }}}{{\tilde{\gamma }}}\right) , \quad \forall z-b<{{\tilde{z}}}<-b. \end{aligned}$$

Then (5.36) is bounded from below by

5.38 $$\begin{aligned}{} & {} \epsilon (t) \int _{ut-1}^{ut} \frac{{\mathrm e}^{-\frac{(ut+z-y)^2}{2s}}}{\sqrt{2\pi s}} {\mathrm e}^{({\tilde{\gamma }}-{\tilde{\beta }})s-{\tilde{\gamma }}{\delta }\left( 1-\frac{{\tilde{\beta }}}{{\tilde{\gamma }}}\right) } {\mathbb {P}}\left[ U_s^b =0 \right] \nonumber \\{} & {} \quad \ge \epsilon (t) \frac{{\mathrm e}^{-\frac{z^2}{2s}}}{\sqrt{2\pi s}} {\mathrm e}^{(1-{\delta })({\tilde{\gamma }}-{\tilde{\beta }})s} \left( 1-{\mathrm e}^{-b^2/2s}\right) , \end{aligned}$$

where we used (4.61) to bound ${\mathbb {P}}\left[ U_s^b =0 \right]$. Setting $s=c\ln (\epsilon (t)^{-1}),$ the lower bound in (5.38) becomes

5.39 $$\begin{aligned} \frac{{\mathrm e}^{\frac{z^2}{2c\ln \left( \epsilon (t)\right) }}}{\sqrt{2\pi c\ln \left( \epsilon (t)^{-1}\right) }} \left( \epsilon (t)\right) ^{1-(1-{\delta })({\tilde{\gamma }}-{\tilde{\beta }})c} \left( 1-{\mathrm e}^{\frac{b^2}{2c\ln \left( \epsilon (t)\right) }}\right) . \end{aligned}$$

Choosing *c* large enough, (5.39) contradicts Assumption (5.37). Hence, the claim of the lemma follows. $\square$

#### Proof of Theorem 2.1

Theorem 2.1 follows directly from Lemmata 5.3, 5.4, and 5.34 in the case $u_c>\sqrt{2\left( {\tilde{\gamma }}-{\tilde{\beta }}\right) }$. The analogous results when $u_c=\sqrt{2\left( {\tilde{\gamma }}-{\tilde{\beta }}\right) }$ are left to the reader. $\square$

## Discussion

In this paper we have used the Feynman–Kac representation to derive the speed of advance of a hitch-hiking subpopulation within an advancing population. Apart from the fact that this allowed fairly sharp control of the precise behaviour of the wave fronts, the method provides a very clear intuitive understanding of the reason for the acceleration in an advancing population compared to a fully established one. Namely, the acceleration is driven by rare excursion of a Brownian bridge reaching ahead of the *B*-population. Translating this back into an underlying individual based model, heuristically this may be interpreted as having excursions of *A* particles into the empty space ahead of the bulk wave taking advantage of higher growth rate in the absence of competition.

Technically, we took advantage of the special features of the model that allowed to reduce the analysis to that of a scalar F-KPP equation with time-dependent parameters. This is a delicate property that gets spoiled already if the diffusion coefficients of the two types are different. An explicit useable Feynman–Kac representation for systems of pdes does not exist. Still, we are optimistic that the Feynman–Kac representation (used for each one-dimensional component of the system) can be used in such situations. This is subject of ongoing research.

## Appendix A: The Laplace method with prefactor

To evaluate the asymptotic behaviour of integrals, we use Laplace’s method. Below we state for convenience the results we use. Proofs can be found in many places in the literature, e.g. Fedoryuk (1989).

### Lemma A.1

Let $f:(0,1)\rightarrow {\mathbb {R}}$ be a twice differentiable function with a unique maximiser at $s^*$ and $f''(s^*)<0$. Moreover, let *P*(*s*, *t*) be a rational function in *s* and *t*. Then

A.1 $$\begin{aligned} \lim _{t\uparrow \infty }\frac{ \sqrt{-tf''(s^*)} \int _0^1 P(s,t) {\mathrm e}^{t f(s)} ds}{\sqrt{2 \pi } P(s^*,t) {\mathrm e}^{tf(s^*)} }=1. \end{aligned}$$

We also need a similar statement for integrals that get their contribution from the boundary of the domain of integration.

### Lemma A.2

Let $f:(y,1)\rightarrow {\mathbb {R}}$ be a twice differentiable function, which is monotone decreasing. Moreover, let *P*(*s*, *t*) be a polynomially bounded function in *s* and *t* in [*y*, 1].

If, for some ${\delta }\ge 0$, $\lim _{x\downarrow y}(y-x)^{-{\delta }} P(y,t) =c(t,y)$, then

A.2 $$\begin{aligned} \lim _{t\uparrow \infty }(tf'(y))^{1+{\delta }} {\mathrm e}^{t f(y)}\int _y^1 \frac{P(s,t)}{c(t,y)} {\mathrm e}^{-t f(s)} ds = \Gamma (1+{\delta }). \end{aligned}$$

## Footnotes

1 A superficial search for “KPP” in MathSciNet finds over 500 entries since 1967 alone.
